# Infection and depletion of CD4^+^ group-1 innate lymphoid cells by HIV-1 via type-I interferon pathway

**DOI:** 10.1371/journal.ppat.1006819

**Published:** 2018-01-05

**Authors:** Juanjuan Zhao, Liang Cheng, Hongbo Wang, Haisheng Yu, Bo Tu, Qiang Fu, Guangming Li, Qi Wang, Yanling Sun, Xin Zhang, Zhenwen Liu, Weiwei Chen, Liguo Zhang, Lishan Su, Zheng Zhang

**Affiliations:** 1 Research Center for Clinical & Translational Medicine, Beijing 302 Hospital, Beijing China; 2 The Lineberger Comprehensive Cancer Center, Department of Microbiology and Immunology, University of North Carolina, Chapel Hill, North Carolina, United States of America; 3 Research Center for Liver Transplantation, Beijing 302 Hospital, Beijing, China; 4 Key laboratory of Infection and Immunity, Institute of Biophysics, Chinese Academy of Science, Beijing, China; 5 Department of Infectious Diseases, Beijing 302 Hospital, Beijing, China; 6 Department of Immonology, Binzhou Medical University, Yantai, Shandong, China; Emory University, UNITED STATES

## Abstract

Innate lymphoid cells (ILCs) are severely depleted during chronic HIV-1 infection by unclear mechanisms. We report here that human ILC1s comprising of CD4^+^ and CD4^-^ subpopulations were present in various human lymphoid organs but with different transcription programs and functions. Importantly, CD4^+^ ILC1s expressed HIV-1 co-receptors and were productively infected by HIV-1 *in vitro* and *in vivo*. Furthermore, chronic HIV-1 infection activated and depleted both CD4^+^ and CD4^-^ ILC1s, and impaired their cytokine production activity. Highly active antiretroviral (HAART) therapy in HIV-1 patients efficiently rescued the ILC1 numbers and reduced their activation, but failed to restore their functionality. We also found that blocking type-I interferon (IFN-I) signaling during HIV-1 infection *in vivo* in humanized mice prevented HIV-1 induced depletion or apoptosis of ILC1 cells. Therefore, we have identified the CD4^+^ ILC1 cells as a new target population for HIV-1 infection, and revealed that IFN-I contributes to the depletion of ILC1s during HIV-1 infection.

## Author summary

Innate lymphoid cells (ILCs), including ILC1, ILC2 and ILC3 populations, represent a novel cellular family of the immune system and have potentials to produce large amounts of T cell-associated cytokines in response to innate stimulation in the absence of specific antigen stimulation. ILCs have emerged as central players in homeostatic and inflammatory conditions, and correlated with the pathogenesis and progression of multiple human diseases. It is reported that ILCs are depleted in HIV-1 infected patients. However, it is not clear whether HIV-1 can infect ILCs and how ILCs are depleted during HIV-1 infection. Here, we find that ILC1s consist CD4^+^ and CD4^-^ subsets and both are present in various human lymphoid organs. We show that HIV-1 can directly infect CD4^+^ ILC1s. HIV-1 infection leads to activation, depletion and functional impairment of ILC1s in humans and in humanized mice *in vivo*. Blocking IFN-I signaling prevents HIV-1-induced apoptosis of ILC1s both *in vitro* and in humanized mice *in vivo*. Our study reveals the CD4^+^ ILC1 population as a new target for HIV-1 infection and identifies an IFN-I mediated mechanism of ILC1 depletion during chronic HIV-1 infection.

## Introduction

Innate lymphoid cells (ILCs) represent a novel family of cellular subsets that produce large amounts of T cell-associated cytokines in response to innate stimulation in the absence of antigens [1, 2]. Based on the expression of specific transcription factors, cell surface markers and signature cytokines [1, 3, 4], ILCs can be divided into three groups. Group 1 ILCs (ILC1s) have been defined as lineage^-^CD127^+^CD117^-^ cells and can produce interferon (IFN)-γ and depend on T-bet for their functions [5]. Group 2 ILCs (ILC2s) are a population of lineage^-^CD127^+^CRTH2^+^ cells that preferentially produce IL-5 and IL-13 and require GATA3 for differentiation [6]. Group 3 ILCs (ILC3s) are lineage^-^CD127^+^CD117^+^cells that have the potential to produce IL-17 and/or IL-22, and are dependent on RORγt [3, 7]. An increasing number of studies have indicated that ILCs represent a heterogeneous family of cells [8–10]. ILC1s were recently divided into CD4^+^CD8^-^, CD4^-^CD8^+^ and CD4^-^CD8^-^ cell populations, and ILC3s comprise CD62L^+^ naïve cells and HLA-DR^+^ ILC3 subsets [8]. These novel ILC subsets still need to be explored with regard to their functionality and clinical significance in humans.

ILCs have emerged as central players in homeostatic and inflammatory conditions. In particular, changes in the number of ILCs have been found to be associated with the pathogenesis and progression of a number of human diseases including chronic infections and inflammatory diseases [1, 3, 11–13]. For example, IFN-γ production by intraepithelial ILC1s promotes inflammation in mouse models of colitis, and blocking of IFN-γ reduces disease severity [12]. In addition, ILC1s may also contribute to human inflammatory bowel diseases, as their numbers have been found to be higher than normal in patients with Crohn’s disease [5, 12]. Changes in the number and function of ILCs have also been documented during HIV-1 or SIV infection. Further, it has been reported that SIV infection results in persistent loss of IL-17-producing ILCs, especially in the jejunum [14]. NKp44^+^ ILC3s are also rapidly depleted in the intestinal mucosa during acute SIV infection [15]. In HIV-1-infected patients, too, ILCs are found to be severely depleted [16–18]. We have previously demonstrated that in HIV-1/SIV infection, ILC3s are depleted through plasmacytoid dendritic cell (pDC) activation and CD95-mediated apoptosis [17] or TLR signaling [19]. However, it is not clear whether HIV-1 influences ILCs through infection and how ILCs are depleted, especially ILC1s, during HIV-1 infection.

In this study, we first showed that tissue ILC1s, as reported previously in the case of human peripheral blood mononuclear cells (PBMCs) [20], consist of CD4^+^, CD8^+^ and CD4^-^CD8^-^ cells, three populations that widely exist in various lymph organs in human. In addition, we found that CD4^+^ ILC1s exhibit significant differences from CD4^-^ ILC1s with regard to their phenotype, cytokine production and expression profile of transcriptional factors. Thus, we have identified a previously unknown CD4^+^ ILC1 population that serves as a target for HIV-1 productive infection. We showed that HIV-1 can infect, activate and preferentially deplete these CD4^+^ ILC1s. Our data was also indicative of the pathogenic effect of sustained type I interferon (IFN-I) signaling during HIV-1 infection, including depletion of ILC1s.

## Results

### Presence of CD4^+^, CD8^+^ and CD4^-^CD8^-^ ILC1 subsets in various human lymphoid organs and their transcription, phenotypes and functionality

**Fig 1:**
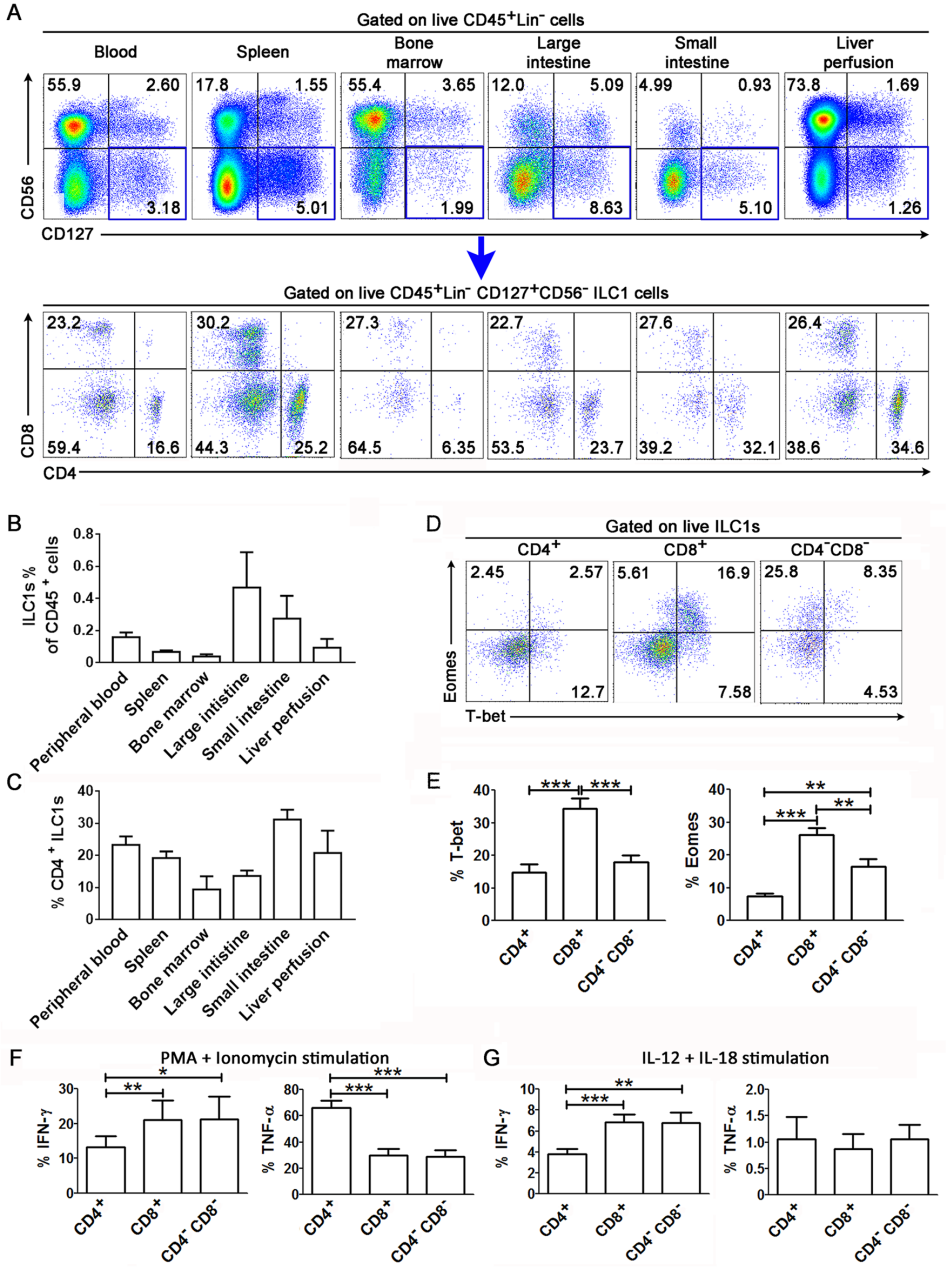
Identification of the CD4^+^, CD8^+^ and CD4^-^CD8^-^ ILC1 subsets in human lymphoid organs. (**A**) The representative dot plots show the tissue distribution of CD4^+^, CD8^+^, and CD4^-^CD8^-^ ILC1 subsets in the peripheral blood, spleen, bone marrow, large intestine, small intestine and liver perfusion. The numbers indicate the percentages of each cell subset. (**B-C**) Summary data of the proportion of total ILC1s in live CD45^+^ cells (**B**) and the proportion of CD4^+^ ILC1s in the total ILC1s (**C**) in various lymphoid organs in humans (n = 25 for PBMCs and n = 5 for other organs). (**D**) Representative dot plots depict the expression of transcriptional factor T-bet and Eomes of CD4^+^, CD8^+^ and CD4^-^CD8^-^ ILC1 subsets in peripheral blood of human. The numbers indicate the percentages of transcriptional factors within each ILC1 subset. (**E**) Summary data of the expression of T-bet and Eomes by ILC1 subsets in peripheral blood of human (n = 15). (**F-G**) Summarized data show the production of IFN-γ and TNF-α by peripheral ILC1 subsets from healthy subjects after stimulation with PMA/ionomycin (n = 12, **F**) and IL-12 plus IL-18 (n = 12, **G**). (**B, C**, **E-G**) Data represent the mean ± s.e.m. values. Overall, *p* < 0.05, one-way ANOVA; *p < 0.05, **p < 0.01, and ***p < 0.001, two-tailed unpaired Student’s *t*-test.

It was recently reported that ILC1s in human peripheral blood contain CD4^+^, CD8^+^ and CD4^-^CD8^-^ subpopulations [20]; however, it is unclear whether these cell populations are present in human lymphoid organs. Here, we investigated the distribution of each ILC1 subpopulation in various human lymph organs. By gating on live human CD45^+^ cells that were negative for lineage-specific surface markers of B cells (CD19 and CD20), T cells (CD3), conventional natural killer (NK) cells (CD16), monocytes and dendritic cells (CD14, CD11c and CD123), and surface markers of hematopoietic precursors (CD34), ILC2 cells (CRTH2) as well as ILC3 cells (CD117), we identified ILC1s as hCD45^+^Lin^-^CD117^-^CRTH2^-^CD127^+^CD56^-^ cells (S1A Fig). Similar to the results of a previous study [20], we found that ILC1s comprise of CD4^+^CD8^-^, CD4^-^CD8^+^ and CD4^-^CD8^-^ subpopulations (S1A Fig). All the ILC1 subsets don’t express the T cell marker TCRαβ, TCRγδ and NK cell marker CD94 which excludes T cell and NK cell contamination; while they express CD5 (S1B Fig). More importantly, we found that the all the three ILC1 subsets, including CD4^+^ ILC1s, were all present in various human lymphoid organs including the spleen, bone marrow, large intestine, small intestine and liver perfusion (Fig 1A). Further analysis indicated that CD45^+^ cells constituted 0.019%–0.818% of the total ILC1 cells (Fig 1B) and CD4^+^ ILC1s constituted 2.35%–39.2% of the total ILC1s in different organs (Fig 1C).

We further investigated the expression of transcriptional factors such as T-bet and eomesodermin (Eomes) in the three ILC1 subsets in human peripheral blood (Fig 1D). We found that CD4^+^ and CD4^-^CD8^-^ ILC1s expressed lower levels of T-bet than CD8^+^ ILC1s (Fig 1E, left). In addition, CD4^+^ ILC1s also expressed lower levels of Eomes than CD4^-^ ILC1 subsets in the blood (Fig 1E, right). We also examined the expression of T-bet and Eomes in ILC1 subsets from various human lymphoid organs by flow cytometry (S2A Fig). We found that in most tissues that we examined, CD4^+^ ILC1s expressed lower levels of T-bet than CD8^+^ or CD4^-^CD8^-^ ILC1s. Notably, the expression levels of T-bet were significantly lower in all ILC1 subsets from the small intestine than in the corresponding subsets from the other organs. This indicates that ILC1s present in the small intestine may have a unique function or activity (S2B Fig). CD4^+^ ILC1s also expressed lower levels of Eomes than CD4^-^ ILC1 subsets in the blood, spleen, bone marrow and liver perfusion. However, the opposite phenomenon was observed in the large and small intestine, where CD4^+^ ILC1s expressed higher levels of Eomes than CD4^-^ ILC1s (S2C Fig). These data suggest that the transcriptional factor profiles of ILC1s differ according to subsets and tissue types. In particular, CD4^+^ ILC1s are characterized by lower expression of T-bet and Eomes transcriptional factors in human peripheral blood.

With regards to phenotypic characteristics, CD4^+^ ILC1s in peripheral blood expressed CD45RA, the NK cell-related molecule CD161, the chemokine receptors CCR6 and CXCR3, death receptor CD95 and adhesive molecule CD11a, which indicate an immature phenotype. However, peripheral blood CD4^+^ ILC1s did not express the integrin CD103; activation markers CD69, CD38 and HLA-DR; proliferation marker Ki67; and death molecules DR5, caspase 1 and caspase 3. Moreover, they expressed low levels of the ILC progenitor marker IL-1R1 (S3 Fig). However, there was no significant difference in the expression of most of the molecules between CD4^+^ and CD4^-^ ILC1 subsets from peripheral blood.

As for functionality, we evaluated cytokine production by peripheral blood ILC1 subsets after PMA/ionomycin or IL-12/IL-18 stimulation (Fig 1F and 1G). We found that CD4^+^ ILC1s produce more TNF-α and lower levels of IFN-γ than CD8^+^ and CD4^-^CD8^-^ ILC1s under PMA/ionomycin stimulation (Fig 1F). Under IL-12/IL-18 stimulation, CD4^+^ ILC1s also produced lower levels of IFN-γ but similar levels of TNF-α than CD8^+^ and CD4^-^CD8^-^ ILC1s (Fig 1G). These ILC1 subsets produced no detectable IFN-γ and TNF-α without stimulation. These data indicate that peripheral blood ILC1 subsets are characterized by functional heterogeneity, and that CD4^+^ ILC1s preferentially produce TNF-α in response to stimulation, as opposed to CD4^-^ ILC1 subsets, which produce lower levels of TNF-α.

Taken together, these comprehensive analyses indicate that CD4+ and CD4- ILC1s exist in various human lymphoid tissues, and their relative numbers, transcription and functionality depend on the subsets and the tissues. In particular, CD4^+^ ILC1s display relatively unique expression of transcriptional factors, immune phenotypes, and cytokine production in relation to CD4^-^ ILC1s.

### *In vitro* and *in vivo* infection of CD4^+^ ILC1s by HIV-1

**Fig 2:**
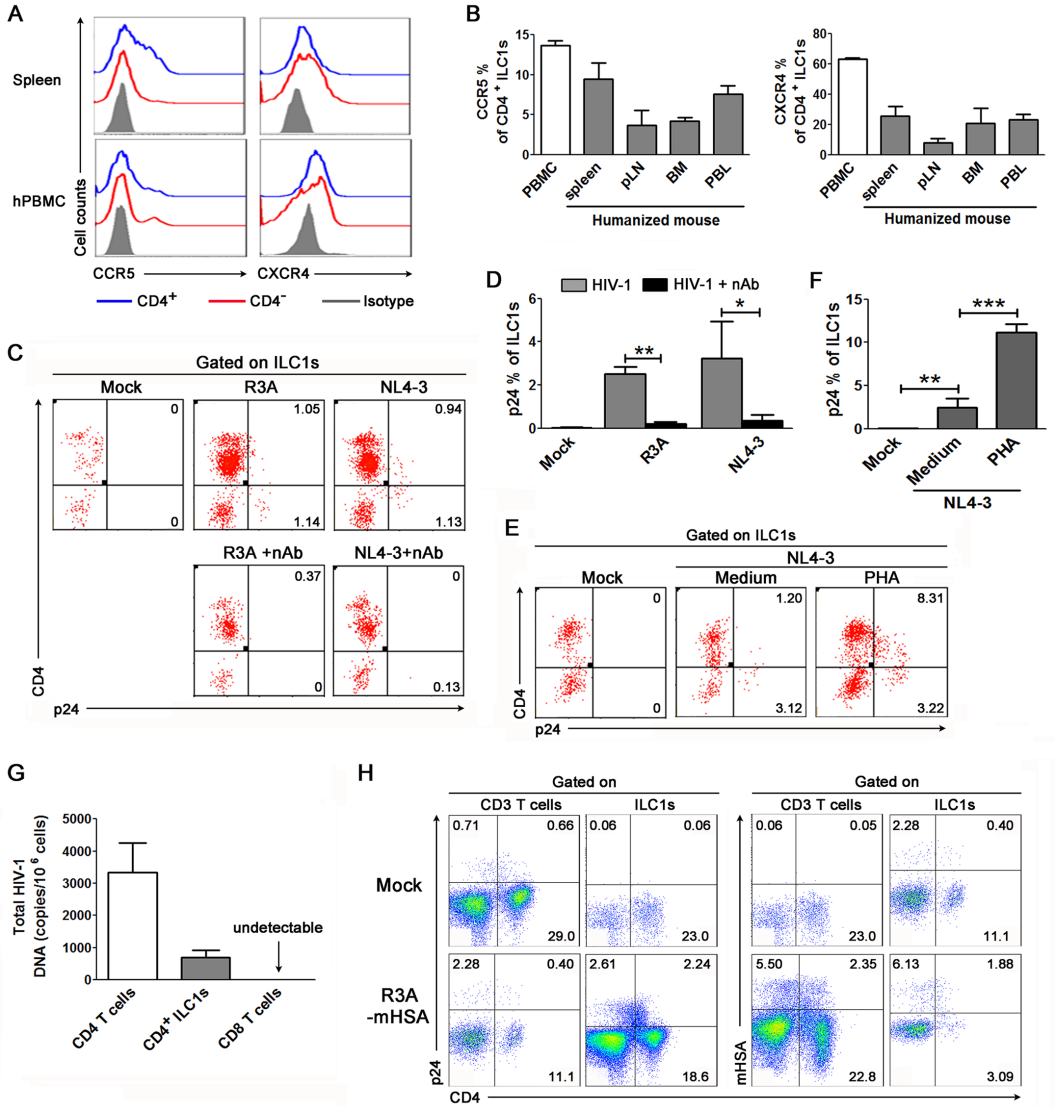
HIV-1 infects ILC1 cells. (**A**) Representative histograms indicate the expression of the HIV-1 co-receptors CCR5 and CXCR4 on CD4^+^ and CD4^-^ ILC1s from the spleen of humanized mouse and human peripheral blood. (**B**) Summary data show the expression of CCR5 and CXCR4 by CD4^+^ ILC1s from human PBMCs (n = 6) and the spleen (n = 14), BM (n = 4), mLN (n = 4) and PBL (n = 3) of humanized mice. BM, bone marrow; mLN, mesenteric lymph node; pLN, popliteal lymph node. (**C-D**) Representative dot plots (**C**) and summarized data (**D**) indicate the p24^+^ ILC1s present *in vitro* of HIV-1 stock. The numbers (**C**) indicate the percentages of p24^+^ cells within ILC1s. Human fresh PBMCs were infected with HIV-1 (R3A and NL4-3) *in vitro* without or with anti-HIV-1 neutralizing antibody. *p < 0.05 and **p < 0.01, two-tailed paired Student’s *t*-test. (**E-F**) Activation enhances HIV-1 infection in ILC1 cells. (**E**) Representative dot plots indicate p24 expression within ILC1s present *in vitro* of mock or HIV-1 NL4-3 stock with or without activation (PHA pre-stimulation for 24 hours). (**F**) Summarized data indicate the percentages of p24^+^ cells within ILC1s in various conditions. Human PBMCs were first incubated with PHA for 24 hours in the presence of IL-2 (50 IU/ml) and IL-7 (20 ng/ml). The cells were then incubated with HIV/NL4-3 stock or mock stock for additional 4 days. **p < 0.01 and ***p < 0.001, two-tailed paired Student’s *t*-test. (**G**) Summarized data show the levels of cell-associated HIV-1 DNA in purified CD4^+^ T cells, CD4^+^ ILC1s and CD8+ T cells from the PBMCs of HIV-1 infected patients (n = 5) detected by real time-PCR. (**H**) Representative dot plots show that HIV-1 infects ILC1s *in vivo* in humanized mice. The numbers indicate the percentages of p24^+^ mCD24^+^ cells within CD3^+^ T cells or ILC1s. At 3 weeks of HIV-1 infection (R3A-HAS virus), bone marrow cells were stained with surface markers, and this was followed by staining of the HIV-1 p24 protein and mCD24. The numbers indicate the percentage of p24 and mCD24 on CD3 T cells and ILC1s in humanized mice (n = 3). (**B**, **D** and **E**) Data represent the mean ± s.e.m. values.

Since a significant proportion of ILC1s express CD4, the receptor for HIV-1 infection, we investigated whether HIV-1 can infect CD4^+^ ILC1s. First, we examined the expression of the HIV-1 co-receptors CCR5 and CXCR4 on ILC1s by flow cytometry. Both CCR5 and CXCR4 were expressed on CD4^+^ ILC1s from human PBMCs and the spleen of humanized mice (Fig 2A). CD4^-^ ILC1s also expressed comparable levels of CCR5 and CXCR4 (Fig 2A). Further analyses indicated that 12% of human CD4^+^ ILC1s express CCR5, while 60% express CXCR4 (Fig 2B). The expression of CCR5 and CXCR4 was also detected on CD4^+^ ILC1s in lymphoid organs, including the spleen, peripheral lymph node and bone marrow, and peripheral blood from humanized mice, but the expression level was slightly lower than that in human PBMCs (Fig 2B).

We then examined whether HIV-1 can infect human CD4^+^ ILC1s. We infected resting human PBMCs with the CXCR4 tropic virus NL4-3 and the CXCR4 and CCR5 dual-tropic virus R3A *in vitro*. Productive infection by HIV-1 was detected by staining of the HIV-1 protein p24 in ILC1s (Fig 2C) and in CD3^+^ T cells (control cells) (S4A Fig). We found that HIV-1 p24 protein was detected in 2.2% of ILC1s after R3A infection and in 3% of ILC1s after NL4-3 infection (Fig 2D), which was comparable to the p24 levels in CD3^+^ T cells (S4A Fig). A neutralizing monoclonal antibody (Clone CH31) specific to the CD4 binding site [21] blocked both R3A and NL4-3 infection by 90% (Fig 2C and 2D and S4 Fig). We also found that HIV-1 infection down-regulated CD4 expression in ILC1s (Fig 2C), as observed in T cells (S4 Fig). Interestingly, when PBMCs were activated by PHA (Fig 2E and 2F), both ILC1s and T cells were infected at higher levels by HIV-1 *in vitro* (Fig 2E and 2F and S5A and S5B Fig). These results indicate that HIV-1 can productively infect ILC1s via the CD4 receptor.

We also examined whether HIV-1 also infected ILC1s *in vivo* in human patients and in humanized mice. We purified CD4^+^ ILC1s from HIV-1-infected patients and determined the cell-associated HIV-1 DNA level by real-time PCR. On average, we detected 800 copies of cell-associated HIV-1 DNA in one million CD4^+^ ILC1s (Fig 2G). As controls, 3200 copies of HIV-1 DNA were detected in one million CD4^+^ T cells, while no HIV-1 DNA was detected in CD8^+^ T cells (Fig 2G). HIV-1 can effectively infect and replicate *in vivo* in humanized *NOD-Rag2*^*-/-*^*γc*^*-/-*^ (NRG-hu) mice transplanted with human CD34^+^ hematopoietic stem cells [22], a highly relevant model for studying HIV-1 induced pathology *in vivo* [23]. We therefore investigated whether CD4^+^ ILC1s could be directly infected by HIV-1 *in vivo* in humanized mice. At 3 weeks after R3A infection, 4.9% of ILC1s expressed p24, while 2.7% of CD3 T cells were positive for p24 (Fig 2H, left). To exclude the possibility that the p24 protein detected here was from virions taken into cells by endocytosis, we infected humanized mice with an engineered R3A reporter virus which expresses the mouse CD24 gene in the Vpr ORF [24]. We found that 8% of ILC1s expressed the mouse CD24 protein (Fig 2H, right). Taken together, our results show that HIV-1 can productively infect CD4^+^ ILC1s both *in vitro* and *in vivo*.

### Increased activation and proliferation of CD4^+^ and CD4^-^ ILC1s in patients with chronic HIV-1 infection

**Fig 3:**
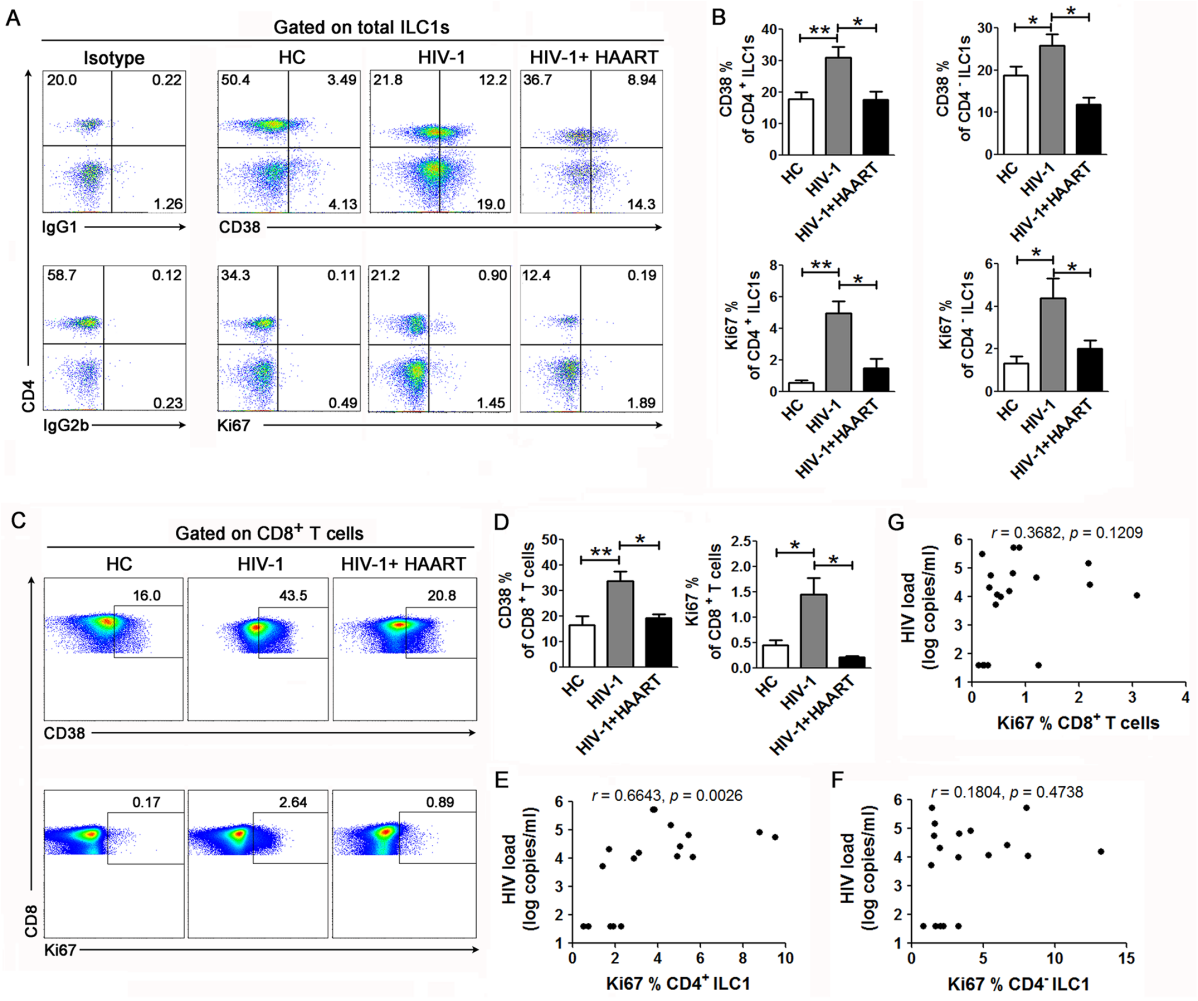
Activation of ILC1s during chronic HIV-1 infection. (**A**-**B**) Representative dot plots (**A**) and summarized data (**B**) show the expression of the activation marker CD38 and proliferation marker Ki67 on CD4^+^ and CD4^-^ ILC1 subsets from the peripheral blood of the healthy donor (n = 18), HIV-1 (n = 30) and HIV-1 plus HAART groups (n = 7). The numbers (**A**) indicate the percentages of each cell subset. (**C**-**D**) Representative dot plots (**C**) and summarized data (**D**) show the expression of the activation marker CD38 and the proliferation marker Ki67 on CD8 T cells from the peripheral blood of the healthy donor (n = 15), HIV-1 (n = 18) and HIV-1 plus HAART groups (n = 7). The numbers (**C**) indicate the percentages of each cell subset. Data represent the mean ± s.e.m. values. Overall, *p* < 0.05, one-way ANOVA; *p < 0.05, **p < 0.01, ***p < 0.001, two-tailed unpaired Student’s *t*-test. (**E-G)** Correlation analysis between Ki67 expression on CD4^+^ (**E**), CD4^-^ ILC1s (**F**) and CD8 T cells (**G**) and plasma HIV-1 load in patients who did not undergo HAART (n = 18). Spearman rank correlation test was used: *r*, correlation coefficient; *p* values are shown.

We next investigated whether HIV-1 infection also activates ILC1s in patients. We analyzed the expression of CD38 and Ki-67 in ILC1s (Fig 3A and S6 Fig). Both CD4^+^ and CD4^-^ ILC1s expressed higher levels of CD38 and Ki67 in HIV-1-infected patients than in the healthy control (HC) subjects, while highly active antiretroviral therapy (HAART) reduced the activation and proliferation of both CD4^+^ and CD4^-^ ILC1s (Fig 3B). As expected, HIV-1 also activated CD8 T cells in HIV-infected patients, and that the activation level was significantly decreased after HAART (Fig 3C and 3D). Further, the percentage of Ki67-expressing CD4^+^ ILC1s, but not CD4^-^ ILC1s, was found to positively correlate with the plasma HIV-1 viral load (Fig 3E and 3F). In contrast, Ki-67 expression in CD8 T cells was not correlated with plasma HIV-1 load in these patients (Fig 3G). These data indicate that HIV-1 infection activated both CD4^+^ and CD4^-^ ILC1s. In particular, the activation of CD4^+^ ILC1s, the HIV-1 target population, was positively correlated with the HIV-1 viral load.

### Preferential depletion of CD4^+^ ILC1s during chronic HIV-1 infection and its correlation with the progression of HIV-1 infection

**Fig 4:**
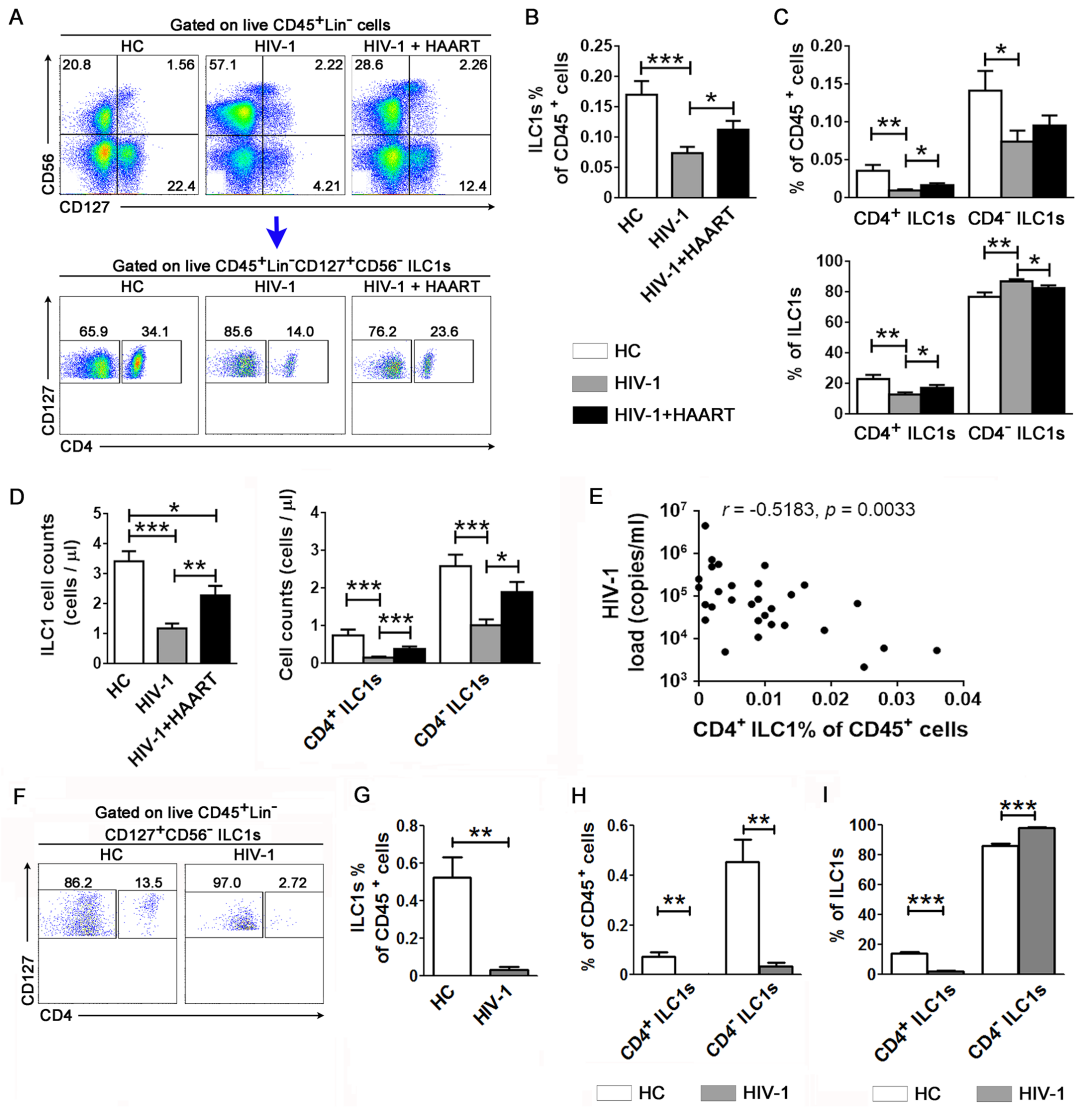
Preferential depletion of CD4^+^ ILC1s during chronic HIV-1 infection and its correlation with disease progression. (**A**) Representative dot plots show the proportion of ILC1 subsets within live CD45^+^lineage^-^ cells in the peripheral blood samples of the HC (n = 26), HIV-1 (n = 30) and HIV-1 plus HAART (n = 12) groups. The numbers indicate the percentages of cell subsets. (**B**) Summarized data show the percentage of ILC1s in CD45^+^ cells. (**C**) Summary data show the percentage of CD4^+^ and CD4^-^ ILC1 subsets in CD45^+^ cells or in total ILC1s in various groups. (**D**) Summary data show the absolute cell counts of total ILC1s, and CD4^+^ and CD4^-^ ILC1 subsets in peripheral blood of various groups. (**E**) Correlation analysis between the CD4^+^ ILC1 percentage in CD45^+^ cells and plasma HIV-1 load. The Spearman rank correlation test is used: *r*, correlation coefficient; *p* values are shown. (**F**) Representative dot plots show the percentage of CD4^+^ ILC1s in the large intestine of a healthy donor and an HIV-1-inected patient. The numbers indicate the percentages of each cell subset. (**G**–**I**) Summarized data show the percentage of ILC1s in CD45^+^ cells (**G**) and the percentage of CD4^+^ or CD4^-^ ILC1 subsets in CD45^+^ cells (**H**) or the total ILC1s (**I**) from the large intestine in HCs (n = 5) and HIV-1-infected patients (n = 4). (**B-D**, **G–I**) Data represent the mean ± s.e.m. values. Overall, *p* < 0.05, one-way ANOVA; *p < 0.05, **p < 0.01, ***p < 0.001, two-tailed unpaired Student’s *t*-test.

We next investigated whether HIV-1 infection depletes ILC1s *in vivo*. Compared to the HCs, ILC1s in CD45^+^ cells were significantly reduced in the peripheral blood of patients with chronic HIV-1 infection (Fig 4A and 4B), and HAART partially reversed the reduction of total ILC1s (Fig 4A and 4B). Further analysis indicated that the percentage of both CD4^+^ and CD4^-^ ILC1s in total CD45^+^ cells was lower in patients with HIV-1 infection than in the HC subjects, while only the CD4^+^ ILC1s but not CD4^-^ ILC1s were significantly rescued by HAART (Fig 4C). The absolute cell counts of total ILC1s and CD4^+^ and CD4^-^ ILC1s were found to be largely reduced in patients with chronic HIV-1 infection as compared to those of HC subjects; and HAART successfully recovered the absolute cell counts of total ILC1s and CD4^+^ ILC1s but not CD4^-^ ILC1s (Fig 4D). Correlation analysis indicated that the percentage of peripheral CD4^+^ ILC1s was negatively correlated with the plasma HIV-1 viral load (Fig 4E) and positively correlated with the CD4/CD8 ratio in the HIV-1-infected subjects (S7 Fig).

We further examined whether ILC1s in the gut were also depleted by HIV-1 infection in humans, which is the key lymphoid organ in HIV-1-associated pathogenesis. As shown in Fig 4F, CD4^+^ ILC1s were significantly depleted in the large intestine in HIV-1-infected patients as compared to the HC donors. The summarized data also showed that the percentage of total ILC1s within CD45^+^ cells was significantly decreased in the large intestine in patients with HIV-1 infection (Fig 4G). Further analysis indicated that the percentage of both CD4^+^ and CD4^-^ ILC1s was reduced during chronic HIV-1 infection (Fig 4H). Importantly, when gated on ILC1 populations, the percentage of CD4^+^ ILC1s was largely decreased and the percentage of CD4^-^ ILC1s was increased accordingly, which indicates that the CD4^+^ ILC1s were preferentially depleted (Fig 4I). These data indicate that CD4^+^ ILC1s from both peripheral blood and large intestine are preferentially depleted during chronic HIV-1 infection.

### Reduced capacity of ILC1 subsets to produce cytokines as a result of persistent HIV-1 infection *in vivo*

**Fig 5:**
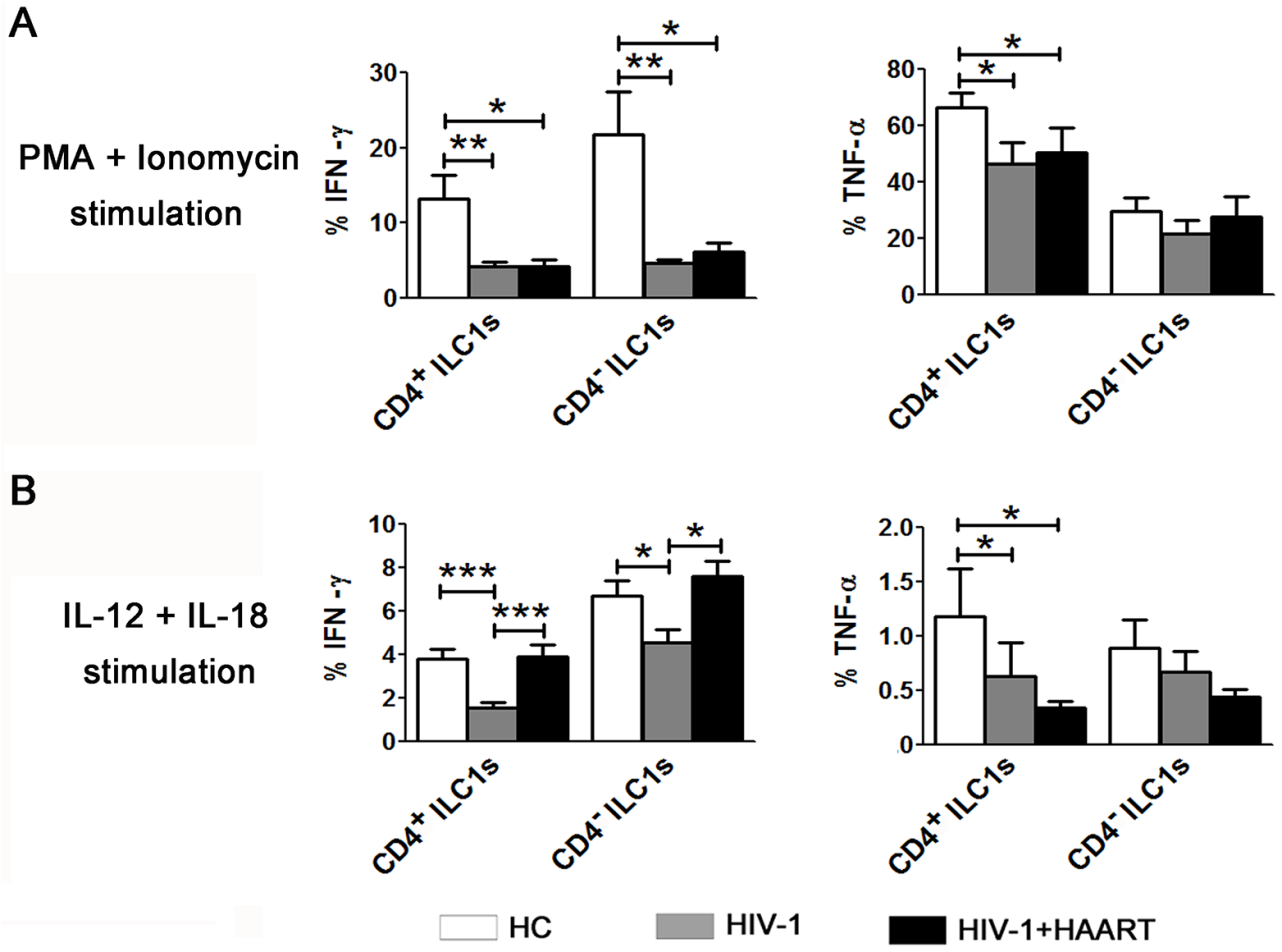
Impairment of cytokine production by ILC1 subsets as a result of chronic HIV-1 infection. Summarized data of IFN-γ and TNF-α production from ILC1 subsets in the peripheral blood of HCs (n = 12), HIV-1-infected patients without HAART (n = 18) and with HAART (n = 12) in response to PMA/ionomycin (**A**) or IL-12 plus IL-18 (**B**). Overall, *p* < 0.05, one-way ANOVA; *p < 0.05, **p < 0.01 and ***p < 0.001, two-tailed unpaired Student’s *t*-test.

ILC1s can produce large amounts of Th1-associated cytokines in response to innate stimulation. We next analyzed whether persistent HIV-1 infection affected the cytokine production ability of ILC1s. As shown in Fig 5A, IFN-γ and TNF-α production by both CD4^+^ and CD4^-^ ILC1 subsets induced by PMA/ionomycin stimulation were significantly lower in HIV-1-infected patients than in HCs. Similar phenomena were also observed when the ILC1s were stimulated by IL-12 and IL-18 (Fig 5B). HAART failed to rescue the function of ILC1 subsets, with the exception that IFN-γ production was rescued by HAART after IL-12 and IL-18 stimulation (Fig 5A and 5B). We thus conclude that chronic HIV-1 infection impaired the ability of the remaining ILC1s, including CD4^+^ ILC1s, to produce cytokines.

### Induction of apoptosis of ILC1s in response to HIV-1 infection

**Fig 6:**
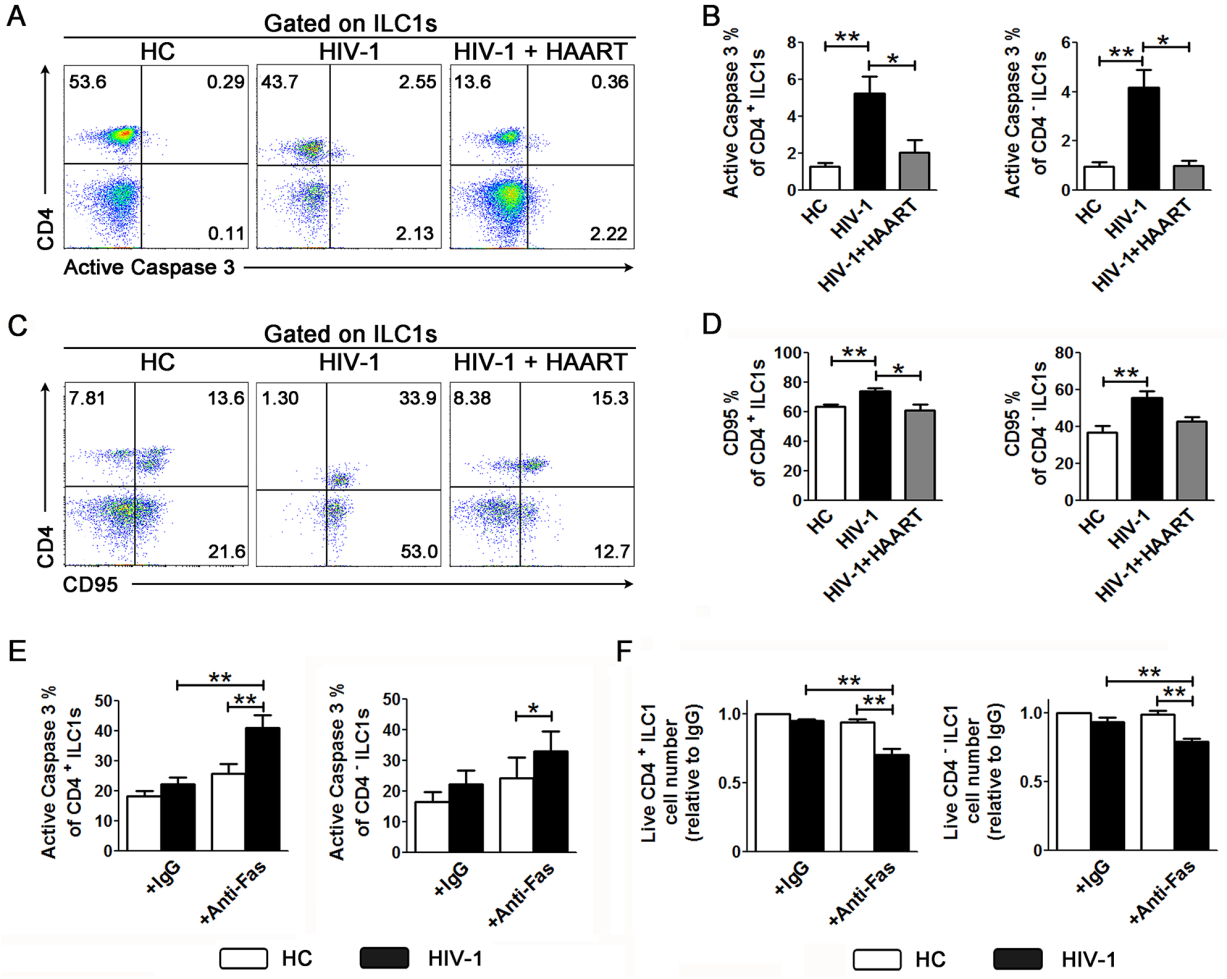
HIV-1 infection leads to ILC1 apoptosis. Representative dot plots (**A** and **C**) and summary data (**B** and **D**) show the percentage of active caspase-3-expressing and CD95-expressing CD4^+^ and CD4^-^ ILC1s in HCs (n = 15), HIV-1-infected patients without HAART (n = 21) and patients with HAART (n = 7). The numbers indicate the percentages of cell subsets (**A** and **C**). Overall, *p* < 0.05, one-way ANOVA; *p < 0.05, **p < 0.01 and ***p < 0.001, two-tailed unpaired Student’s *t*-test (**B** and **D**). (**E** and **F**) Summary data indicate the percentage of active caspase 3 in CD4^+^ and CD4^-^ ILC1s (**E)** and the relative live cell number of CD4^+^ ILC-1s and CD4^-^ ILC1s (**F**) after anti-Fas Ab-induced apoptosis in humans. PBMCs from HCs (n = 5) and HIV-1-infected subjects who did not undergo HAART (n = 5) were pre-incubated with IL-2, IL-12 and IL-18 for 12 h and subsequently stimulated with the plate-bound anti-Fas antibody for 24 h. The cells were then collected, counted and stained to detect active caspase 3 expression. *p < 0.05 and **p < 0.01, two-tailed paired Student’s *t*-test (**E** and **F**).

We next examined how HIV-1 infection leads to ILC1 depletion. We discovered that chronic HIV-1 infection significantly up-regulated active caspase-3 expression in both CD4^+^ and CD4^-^ ILC1s (Fig 6A and 6B). In contrast, caspase1 was not significantly up-regulated in CD4^+^ ILC1s (and only slightly increased in CD4^-^ ILC1s) of patients with HIV-1 infection as compared to the HC subjects (S8A and S8B Fig). HAART could significantly decrease the expression of active caspase-3 in both CD4^+^ and CD4^-^ ILC1s (Fig 6A and 6B), correlated with rescued ILC1s. These findings indicate that HIV-1 infection leads to depletion of ILC1 subsets via apoptosis-dependent mechanisms.

We further investigated whether the Fas/FasL pathway was involved in the apoptosis of ILC1s (up-regulation of active caspase-3), as reported in ILC3s in our previous study [17]. We found that expression of CD95 was significantly up-regulated in both CD4^+^ and CD4^-^ ILC1s from patients with chronic HIV-1 infection compared with HC subjects (Fig 6C and 6D). HAART decreased the expression of CD95 in CD4^+^ but not CD4^-^ ILC1s (Fig 6C and 6D). In contrast, the expression of death receptor 5 (DR5) was not up-regulated in ILC1 subsets in patients with chronic HIV-1 infection (S8C and S8D Fig). Notably, the expression of caspase-3 and CD95 was also up-regulated in CD8^+^ T cells in HIV-1-infected patients as compared to HC subjects (S9A and S9B Fig).

We then investigated whether the Fas/FasL pathway is involved in the apoptosis of ILC1 subsets. After *in vitro* stimulation with the anti-CD95 agonist antibody, both CD4^+^ and CD4^-^ ILC1s from HIV-1-infected patients displayed higher levels of active caspase-3 expression than those from HCs (Fig 6E). Accordingly, the number of live CD4^+^ and CD4^-^ ILC1s was significantly reduced after treatment with the anti-CD95 agonist antibody as compared to the IgG control in HIV-1-infected patients but not in the HC subjects (Fig 6F). Thus, the number of live CD4^+^ and CD4^-^ ILC1s in HIV-1-infected patients was markedly less than that in HC subjects in response to *in vitro* stimulation with the same anti-CD95 agonist antibody (Fig 6F). This indicates that ILC1 subsets from HIV-1-infected patients are more sensitive to Fas/FasL signaling than those from HC subjects. We conclude that the Fas/FasL pathway is actively involved in the apoptosis of ILC1 subsets in patients with chronic HIV-1 infection.

### Role of IFN-I signaling in HIV-1 induced ILC1 depletion

**Fig 7:**
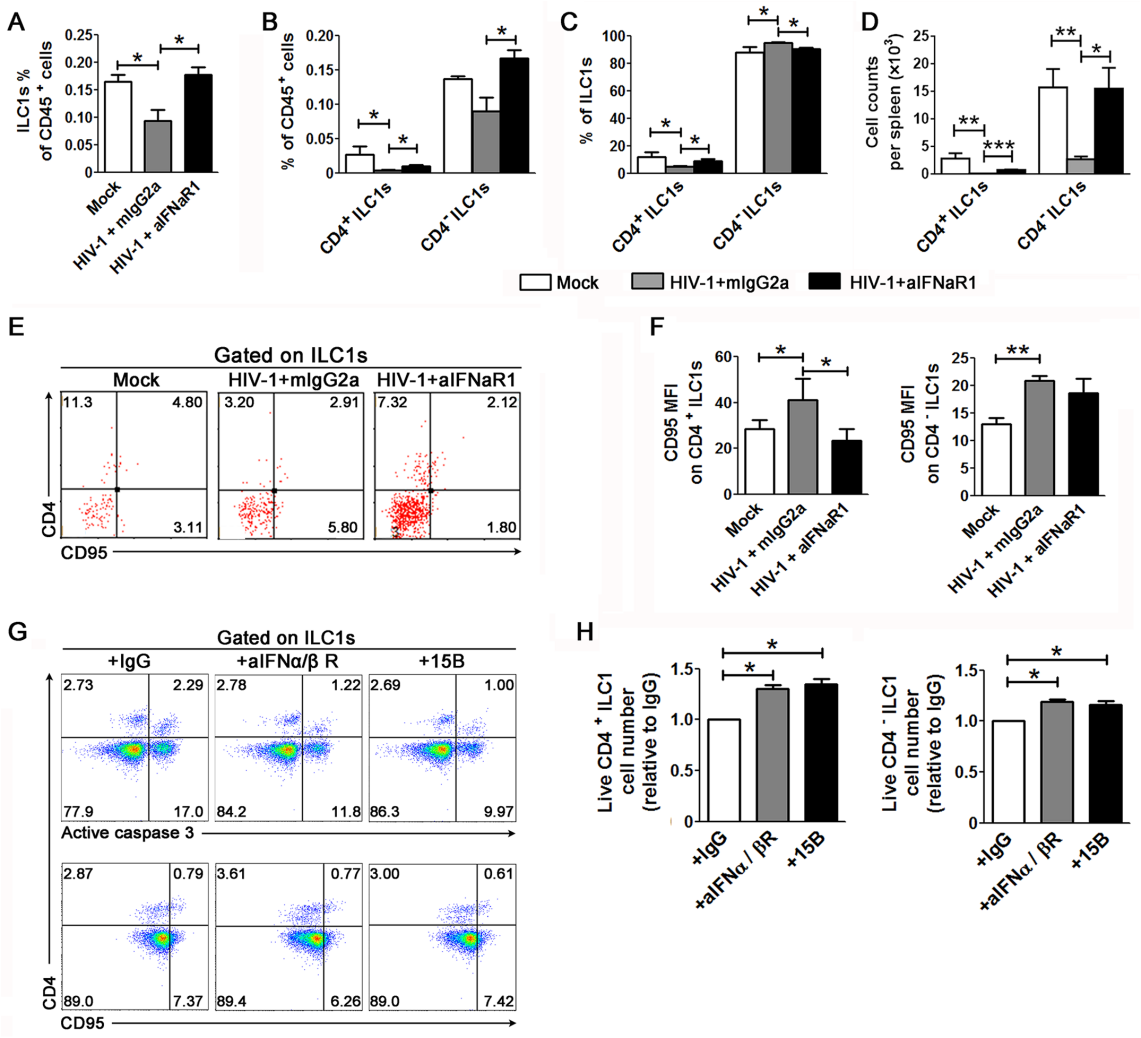
Prevention of ILC1 depletion by blockade of IFN-I signaling. (**A**–**D**) Humanized mice infected with HIV-1 were treated with α-IFNAR mAb or isotype control (mouse IgG2a) twice a week from 6 to 10 weeks after infection. Mice were sacrificed at 10 weeks after infection. (**A**) Summary data show the percentage of ILC1s in the total CD45^+^ cells present in the spleen of humanized mice. (**B-C**) Summary data show the percentage of CD4^+^ and CD4^-^ ILC1s in total CD45^+^ cells (**B**) and in ILC1s (**C**) present in the spleen of humanized mice, respectively. (**D**) Absolute number of CD4^+^ and CD4^-^ ILC1s in the spleen of humanized mice. (**E-F**) Representative dot plot (**E**) and summarized data (**F**) show CD95 expression on ILC1s in the spleen of humanized mice. (**A–D, F**) Mock, n = 3; HIV-1 plus mIgG2a, n = 5; HIV-1 plus α-IFNAR, n = 5. Data are representative of three independent experiments with three donors. Data represent the mean ± s.e.m. values. *p < 0.05, **p < 0.01, ***p < 0.001, two-tailed unpaired Student’s *t*-test. (**G** and **H**) PBMCs from HIV-1-infected patients were incubated with IL-12 and IL-18 for 12 h, and were then incubated with the isotype control antibody (IgG), immunotoxin 15B-sap or anti-IFNα/β receptor antibodies for an additional 72 h. (**G**) Representative dot plots show the percentage of active caspase-3- and CD95-expressing CD4^+^ and CD4^-^ ILC1 subsets *in vitro* with 15B and anti-IFNα/βR antibody incubation. The numbers indicate the percentage of cell subsets. Data are representative of three independent experiments with four donors. (**H**) Summarized data show the number of live CD4^+^ and CD4^-^ ILC1s after culture *in vitro* with 15B and anti-IFNα/βR antibodies.

Sustained IFN-I signaling has been reported to be correlated with and contribute to SIV and HIV-1-induced immune pathogenesis [25–27]. We have proved that depletion of pDCs or blocking IFN-I signaling prevents HIV-1-induced T cell and ILC3 depletion *in vivo* [17, 26, 28]. We thus investigated whether IFN-I signaling also contributes to HIV-1-induced ILC1 depletion *in vivo*. We treated HIV-1-infected humanized mice with the anti-IFNAR1 mAb [26] from week 6 through week 10 after infection. At 10–12 weeks after infection, we terminated the mice and measured ILC1 number and phenotype in each group. We found that blockade of IFN-I signaling with the anti-IFNAR1 mAb rescued both CD4^+^ and CD4^-^ ILC1s cells in percentages (Fig 7A–7C) and in numbers (Fig 7D) as compared to the isotype IgG control group. In addition, we found that blocking the IFN-I pathway significantly decreased CD95 expression on CD4^+^ ILC1s in humanized mice with persistent HIV-1 infection (Fig 7E and 7F). We further cultured PBMCs from HIV-1-infected patient *ex vivo* in the absence or presence of pDC-depleting 15B mAbs conjugated with the SAP toxin (immune toxin 15B-sap) or the anti-IFNα/β receptor blocking antibody. We observed significant downregulation of both CD95 and active caspase-3 expression in CD4^+^ ILC1s from HIV-1-infected patients cultured *in vitro* in the presence of the immune toxin 15B-sap or anti-IFN-α/β receptor antibodies as compared to the IgG control (Fig 7G). Therefore, depletion of pDCs or blockade of IFNAR1 both prevents HIV-1 induced ILC-1 depletion *in vitro* (Fig 7H). These data indicate that IFN-I signaling contributes to ILC1 depletion during chronic HIV-1 infection.

Since HAART fails to restore ILC function in HIV-1-infected patients, we therefore investigated whether blocking IFN-I signaling combined with combined antiretroviral therapy (cART) *in vivo* can rescue the function of ILC1s in HIV-1 infected humanized mice. We treated HIV-1 infected mice with cART at 4 weeks post infection (wpi). As reported [26], 3 week after cART, the infected humanized mice received α-IFNAR1 mAb treatment for 3 weeks from 7 to 10wpi. The function of ILC1 was analyzed at 12wpi. Interestingly, we found that cART alone restored IFN-γ and TNF-α production by splenic ILC1s under PMA/Ionomycin stimulation (S10B Fig). IFN-I blockade in combination with cART did not further increase IFN-γ and TNF-α production by splenic ILC1s (S10B Fig). The result is different from human studies which indicated that HAART cannot rescue ILC1 function (Fig 5). One possible reason for the differences is that we started cART treatment in humanized mice at early infection phase (4 weeks post HIV-1 infection), while in HIV-1 infected patients HAART is usually initialized years after infection at chronic phase of the infection included in our study. Further studies are needed to unveil the effect of IFN-I signaling on ILC1 function.

## Discussion

Our study investigates the heterogeneity of ILC1s in human lymphoid organs, and provides the first piece of evidence to show that HIV-1 can directly infect CD4^+^ ILC1s and lead to their activation, depletion and functional impairment *in vivo* in humans and in humanized mice. Successful HAART rescued the number of CD4^+^ ILC1s but not cytokine production activity via the inhibition of Fas/FasL-mediated apoptosis of ILC1s. This study, therefore, is the first to identify CD4^+^ ILC1s as important HIV-1 target cells, and may serve as a novel target of HIV-1 therapies aimed at human immune reconstitution.

Through a comprehensive analysis of lymphocytes from human spleen, bone marrow, large intestine, small intestine and liver, we found that human ILC1s consist of CD4^+^, CD8^+^ and CD4^-^CD8^-^ populations and that all these populations are widely present in all lymphoid organs, which has not been described in previous studies [8, 9, 20]. We also observed that CD4^+^ ILC1s expressed immature phenotypes and lower levels of Th1-associated transcriptional factor T-bet and Eomes than CD4^-^ ILC1s and higher level of TNF-α in response to stimulation. It Is not clear where these CD4+ ILC1s are developed and how the immature ILC1 subsets traffic to various lymphoid tissues. A recent study based on mass cytometry- and t-SNE-based analysis showed ILC1s were undetectable across different human tissues [10]. However, in a re-analysis on CyTOF dataset, ILC1s are clearly clustered in lymphoid tissues [29]. Another report also suggested that ILC1s reported in previous studies may be attributable to CD5^+^ T cell contamination [30]. However, CD5 is also expressed and functions independently of T cells [31]. Indeed, ILC1s express high levels of CD5 in our study and previous report [32]. Therefore, the use of CD5 with CD4 or CD8 in ILCs without confirming surface CD3 or TCR expression does not definitively identify CD4^+^ and CD8^+^ T cells [20]. Furthermore, human patients with RAG1 deficiency, who lack T cells, are characterized by the presence of circulating ILC1s at frequencies comparable to those of ILC2s and ILC3s [33]. ILC1s have also been cloned under T-cell-promoting conditions, and have been detected in inflamed intestinal tissues of patients suffering from Crohn’s diseases [5, 34]. Taken together, our data provide a comprehensive description of the heterogeneity of CD4^+^ and CD4^-^ ILC1s (not T cells) across various lymphoid tissues in humans.

The identification of CD4 expression on ILC1s led to the question of whether this population can be infected by HIV-1. Our results clearly showed that CD4^+^ ILC1s also express CCR5 and CXCR4 and can be productively infected by HIV-1 both *in vitro* and *in vivo*. The relative infection and replication of HIV-1 in CD4^+^ ILC1s is comparable to that in CD4 T cells. Interestingly, PHA activation of PBMC enhanced HIV infection in both CD4+ ILC1 and T cells. These results indicate that CD4^+^ ILC1s are HIV-1 target cells and possibly support HIV-1 persistence in patients with chronic HIV-1 infection. Therefore, we identified CD4^+^ ILC1s as a new target for HIV-1 infection. Further studies to identify whether CD4^+^ ILC1s serve as an HIV-1 reservoir in HIV patients during HAART will be important for developing strategies for HIV-1 treatment.

It has been reported that HIV-1 infection leads to depletion of all ILC subsets, including ILC1s, in circulation [16, 17, 19] and lymphoid organs [18]. We discovered here that HIV-1 infection also depleted ILC1s in the large intestine of patients. Unlike the results of a previous study [16], we found that HAART can rescue the number of peripheral ILC1s in HIV-1-infected patients. This discrepancy could be explained by the difference in the cohorts enrolled in the two studies. Differences in the time of HAART onset may lead to differences in immune reconstitution [35, 36], which may then affect the restoration of the number of ILC1s. Our results indicate that HIV-1 infection depletes ILC1s both in circulation and in lymphoid organs. Of particular note, we found that CD4^+^ ILC1s were preferentially depleted within the total ILC1 population, which indicates that they are more sensitive to HIV-1-induced apoptosis.

The mechanism underlying HIV-1-induced depletion of ILC1s is poorly defined. We have reported previously that HIV-1 infection induces depletion of ILC3s via Fas/FasL signaling in a pDC/IFN-I-dependent manner [17]. In the present study, we found that the depletion of ILC1s was also associated with cell apoptosis mediated by the Fas/FasL pathway during HIV-1 infection. We therefore tested the pDC/IFN-I axis in humanized mice with HIV-1 infection. Our data clearly showed that blocking IFN-I signaling with an antibody against IFNAR1 prevented HIV-1-induced depletion of ILC1s *in vivo* in humanized mice. Furthermore, blocking IFN-I signaling or depletion of pDCs during *in vitro* culture of PBMCs from HIV-1 infected patients also significantly reduced ILC1 apoptosis and rescued their number. We thus have demonstrated that pDC and IFN-I signaling plays a critical role in ILC1 depletion during chronic HIV-1 infection.

Our data demonstrate that HIV-1 infection not only depletes ILC1s but also leads to their activation and functional impairment, as indicated by the significant decrease observed in their production of cytokines, including IFN-γ and TNF-α. Interestingly, HAART rescues ILC1s in number but fails to recover their function of cytokine production in HIV-1-infected patients. In HIV-1-infected humanized mice, however, we found that HAART starting during early phase of infection (4wpi) rescued both ILC1 number and functions in IFN-γ and TNF-α production. This differential effect of HAART on ILC1 function may be due to different treatment time in patients and in humanized mice. For patients in the study, HAART was usually initialized years after HIV-1 infection at chronic infection phase; while HAART was given at early phase of HIV-1 infection (4 weeks) for humanized mice in the study. Indeed, our findings are similar to a previous report in which antiretroviral therapy initialized during acute infection could preserve ILCs in patients [16]. These data also indicate that depletion of ILC1s and their functional impairment may be mediated by various mechanisms during short acute and long chronic infection. We have recently found that pDC depletion or blockade of IFN-I signaling could significantly reduce residual immune activation and restore anti-HIV immunity in HIV-1-infected humanized mice without or with cART [26]. Future studies should focus on the differential mechanisms underlying cell depletion and functional impairment of ILC1 subsets, and determine whether HAART combined with IFN-I blockade can restore ILC1 function in chronic HIV-1 infection in human patients.

In summary, we identified subset- and tissue-dependent heterogeneity of ILC1s and provided evidence to show that CD4^+^ ILC1s are a novel target for HIV-1 infection. Further, we demonstrated that IFN-I signaling contributes to the depletion of ILC1s, at least partly through the Fas/FasL pathway during HIV-1 infection. These new findings, therefore, extend our earlier findings which show that sustained pDC activation and IFN-I production contributes to HIV-1 pathogenesis. Therefore, blockade of the pDC/IFN-I axis will be a novel therapeutic stratagem to reverse HIV-1-induced pathogenesis, including ILC1 depletion and impairment.

## Materials and methods

### Ethics statement

Approval for animal work was obtained from the University of North Carolina Institutional Animal Care and Use Committee (IACUC ID: 14–100). The study protocol on human samples was approved by the Institutional Review Board and the Ethics Committee of Beijing 302 Hospital in China. The written informed consent was obtained from each subject. All samples were anonymized in the study. Human tissue samples, including the spleen, small intestine, large intestine, bone marrow and liver perfusion, used in this study were obtained from adult donors who had undergone liver transplantation as healthy controls. Gut mucosa from HIV-1-infected patients were obtained for pathological diagnosis. Written informed consent was obtained from each donor. Complete RPMI media were used for all cell isolation experiments. Human fetal livers and thymuses (gestational age 16 to 20 weeks) were obtained from medically indicated or elective termination of pregnancies through a non-profit intermediary working with outpatient clinics (Advanced Bioscience Resources, Alameda, CA). Written informed consent from the maternal donor was obtained in all cases under regulations governing the clinic. All animal studies were conducted following NIH guidelines for housing and care of laboratory animals. The project was reviewed by the University’s Office of Human Research Ethics, which determined that this submission does not constitute human subjects research as defined under federal regulations [45 CFR 46.102 (d or f) and 21 CFR 56.102(c)(e)(l)].

### Patients

Thirty HIV-1-infected HAART-naïve individuals and 12 HIV-1-infected patients who underwent successful HAART were enrolled in our study (S1 Table). The majority of these individuals had been infected with HIV-1 via sexual transmission, while a few subjects were paid blood donors. Twenty-six uninfected subjects were employed as healthy controls (HCs). The study protocol was approved by the Ethics Committee of Beijing 302 Hospital, and written informed consent was obtained from each subject.

### Human tissue cell isolation

Immune cells from human samples were isolated according to previously reported protocols. In brief, peripheral blood mononuclear cells (PBMCs) and bone marrow cells were isolated by Ficoll-Hypaque density gradient centrifugation of heparinized blood of enrolled subjects. The spleen was first ground on ice, after which the cells were collected and filtered. The liver perfusion was directly filtered and concentrated by centrifugation (750 g, 15 min, 20°C), and was layered onto the Ficoll gradient. The small intestine and large intestine were first finely minced using scalpels, and were then incubated with 0.8 mg/mL collagenase type IV (Worthington-Biochemical) and DNase I (Roche) for 1 h before they were filtered through a 70-mm strainer. The filtered cells were collected and isolated in a similar manner to PBMCs. Upon isolation, all the cells were cryopreserved in 90% fetal calf serum plus 10% DMSO for subsequent assay.

### Construction of humanized mice

We constructed NRG-hu mice using a previously reported method [22]. Briefly, human CD34^+^ cells were isolated from 16- to 20-week-old fetal liver tissues (Advanced Bioscience Resources, Alameda, CA). The tissues were digested with liver digest medium (Invitrogen, Frederick, MD). The suspension was filtered through a 70-μm cell strainer (BD Falcon, Lincoln Park, NJ) and centrifuged for 5 min to isolate mononuclear cells by Ficoll gradient centrifugation. After selection with the CD34^+^ magnetic-activated cell sorting (MACS) kit, CD34^+^ hematopoietic stem cells were injected into the liver of each irradiated (300 rad) 2- to 6-day-old NRG mouse (0.5 × 10^6^/mouse). More than 95% of the humanized mice were stably reconstituted with human leukocytes in the blood (60%–90% at 12–14 weeks). The level of engraftment was similar in each cohort. All the mice were housed at the University of North Carolina at Chapel Hill.

### Tissue processing of humanized mice

Total leukocytes were isolated from the spleen of humanized mice as previously described [22]. Lymphoid tissues, including red blood cells, were lysed with the ACK buffer, and the leukocytes were stained and fixed with 1% formaldehyde before FACS analysis. The total cell number was quantified by Guava Easycytes with the Guava Express software.

### HIV-1 virus stocks and infection of humanized mice

An R5-tropic strain of HIV-1, JR-CSF (NIH AIDS reagents program, Cat# 2708), was used for inducing persistent HIV-1 infection. Viruses were generated by transfection of 293T cells (SIGMA-ALORICH, Cat# 12022001-1VL). R3A-HSA was constructed by replacing the vpr gene with mouse heat stable antigen (HSA; CD24) as reported previously. Humanized mice with stable human leukocyte reconstitution were infected with JR-CSF or R3A-HSA at a dose of 10 ng p24/mouse, through an intra-orbital injection. Humanized mice infected with mock-transfected 293T cell culture supernatant were used as control groups. For acute HIV-1 infection, viral genomic RNA present in the plasma was measured by real-time PCR (ABI Applied Biosystem). An X4 and R5 dual-tropic strain of HIV-1, R3B/Av1v2, was used for the *in vitro* experiment.

### *In vitro* HIV-1 infection

Fresh PBMCs were incubated with the infectious HIV-R3A stock, NL4-3 stock or mock stock with or without the neutralizing monoclonal antibody (Clone CH31) for 2 h at 37°C. Then, the cells were incubated in complete RPMI 1640 medium at a density of 2 × 10^6^ cells/ml in the presence of IL-2 (50 IU/ml) and IL-7 (20ng/ml) for an additional 3 days. Alternatively, fresh PBMCs were activated with phytohemagglutinin (PHA, 5 μg/ml) or medium in the presence of IL-2 (50 IU/ml) and IL-7 (20 ng/ml) for 24 hours. Then the cells were incubated with the infectious NL4-3 stock or mock stock for an additional 4 days. Intracellular p24 expression on ILC1 subsets or CD3^+^ T cells was determined by flow cytometry as described above.

### Blockade of human IFN-I signaling in NRG-humanized mice

An anti-IFNAR1 blocking antibody was developed as per our recent report [26]. Briefly, the human IFNAR1 expression cell line 293T was first incubated with the supernatant of the hybridoma and then incubated with the PE-labeled goat anti-mouse IgG secondary antibody. Then, an IFN-I reporter cell line 293T stably transfected with a mouse A2 promoter-driven EGFP was used to screen antibody clones that could block human IFNAR1 signaling. Humanized mice with HIV-1 infection were treated intraperitoneally with anti-IFNAR1 blocking antibodies from 7 to 10 weeks post-infection twice a week at a dose of 400 μg/mouse at the first treatment and 200 μg/mouse for the following treatments. The same dose of mouse isotype IgG2a control was used in all the experiments. Alternatively, the HIV-1-infected mice were treated with combination antiretroviral therapy (cART) as reported [26]. HIV-1 infected, cART treated mice were treated i.p. with IFNAR1 blocking antibodies from 7 to 10 wpi twice a week with 400 μg/mouse at the first injection and 200 μg/mouse for the following treatments. A same dose of mouse isotype IgG2a control was use in all experiments.

### Flow cytometry

Flurochrome-conjugated antibodies or regents obtained from Biolegend, BD Bioscience, eBioscience and R&D Systems were used in the study. Live/dead fixable violet dead cell dye (LD7) was purchased from Molecular Probes (Eugene, OR). For humanized mice, live human leukocytes (Y7^-^mCD45^-^hCD45^+^) were analyzed for ILC1 subsets and other cell subsets or phenotypes with CyAn FACS (Dako, Beckman Coulter, Denmark). The data were analyzed with the Summit Software. For human PBMCs and various tissue-derived lymphocytes, dead cells were excluded using the fixable viability dye eFluor 450 (eBioscience). The remaining live CD45^+^ cells were analyzed for phenotypic expression with FACS CANTO II, and the data obtained were further analyzed with the FlowJo software (TreeStar, San Carlos, CA). Cytokines, including IL-2, IL-12 and IL-18, were purchased from PeproTech (Rocky Hill, NJ).

For surface marker staining, leukocytes were incubated with antibodies on ice for 30 min and then washed and fixed for further analysis. For staining of HIV-1 gag p24, transcriptional factors, Ki67 and the apoptotic marker active caspase-3, the cells were stained with the surface marker first, and then permeabilized using a Cytofix/Cytoperm kit (BD Bioscience) and stained for intracellular protein. Alternatively, fresh cells were mixed with caspase-1 for 2 h for caspase-1 staining and were then subjected to surface staining.

For intracellular cytokine detection, freshly isolated cells were stimulated for 6 h by culturing with PMA (50 ng/ml, Sigma) and ionomycin (1 μM, Merck) in the presence of BFA (1 μM). Alternatively, the cells were incubated with IL-12 (20 ng/ml) plus IL-18 (20 ng/ml) for 12 h, followed by Golgi-stop for an additional 6 h. The cells were then collected for surface marker staining; this was followed by cell permeabilization and intracellular cytokine staining. For CD107a staining, the cells were incubated with anti-CD107a antibodies from the onset of stimulation. Then, the cells were further incubated with BFA for an additional 6 h.

### Cell sorting and cell-associated HIV-1 DNA detection

Freshly isolated PBMCs from HC and HIV-1-infected patients were enriched for ILCs by depletion of CD3^+^ T cells, CD14^+^ monocytes and CD19^+^ B cells using microbeads (Miltenyi Biotech, Germany). Then, the enriched cells were sorted on a FACSAria II (BD Biosciences). CD4^+^ ILC1s were isolated by sorting on live cells, singlets, scatter, and lineage^-^CD56^-^CD127^+^CD4^+^ cells (lineage including CD3, CD14, CD16, CD19, CD34, CD11c, CD123, CD117 and CRTH2). CD4^+^ and CD8^+^ T cells were directly sorted from PBMCs. Then, nucleic acid was extracted by sorting CD4^+^ ILC1s, CD4^+^ T cells and CD8^+^ T cells using the DNAeasy minikit (Qiagen) to measure total cell-associated HIV-1 DNA. HIV-1 DNA was quantified by real-time PCR according to our previous protocol. DNA from serial dilutions of ACH2 cells, which contain 1 copy of the HIV-1 genome per cell, was used to generate a standard curve.

### Apoptosis assays

Frozen PBMCs from HCs and HIV-1-infected patients were thawed and cultured in complete RPMI (RPMI 1640 containing 10% heat-inactivated fetal bovine serum, 2 mM l-glutamine, 100 U/ml penicillin and 100 mg/ml streptomycin sulfate) (Cellgro, Manassas, VA) with IL-12 (10 ng/ml), IL-18 (10 ng/ml) and IL-2 (50 IU/ml) for 12 h. Then, the cells were collected to perform *in vitro* assays. The cells were incubated in the presence of plate-bound anti-CD95 monoclonal antibody or isotype control antibody (5 μg/ml, clone CH11, Millipore) for an additional 24 h. Alternatively, the cells were incubated with 15B mAb conjugated with the toxin sap (15B-sap, 8 ng/ml) to deplete pDCs or with anti-IFN-α/β receptor antibodies (10 μg/ml, Millipore) to block IFN-I signaling for an additional 72 h. Then, the cells were harvested, and the number of live cells was counted and stained for active caspase-3 and/or CD95 expression by ILC1 subsets.

### Statistical analysis

Data were analyzed using GraphPad Prism software version 5.0 (GraphPad software; San Diego, CA, USA). The data represent the mean ± s.e.m values. One-way ANOVA was used for primary comparisons between different groups, and the result was represented by the overall *p* value. Secondary comparisons between any two different cohorts of mice or patients were performed using a two-tailed unpaired Student’s *t*-test. Correlations between variables were evaluated using the Spearman rank-correlation test. Results were considered significant at *p* values <0.05.

## Supporting information

S1 Table Characteristics of the study participants. (DOCX)

S1 Fig Gating strategy for peripheral blood ILC1 subsets. **(A)** Representative dot plots identify ILC1 subsets. After gating on lymphocytes (FSC-SSC), singlets, and live CD45^+^ cells, the cells that remained were identified as lineage^-^CD127^-^CD56^+^ NK cells and lineage^-^CD127^+^CD56^-^ total ILC1 cells. Based on CD4 and CD8 expression, ILC1s were further divided into CD4^+^CD8^-^, CD4^-^CD8^+^ and CD4^-^CD8^-^ ILC1s, while the NK cells did not contain any CD4^+^CD8^-^ cell subpopulations. The lineage markers included CD3, CD14, CD16, CD19, CD123, CD11c, CD34, CD117 (excluding ILC3) and CD294 (CRTH2, excluding ILC2). The numbers indicate the percentage of cell subsets. **(B)** A representative histogram shows the expression of TCRαβ, TCRγδ, CD5 and CD94 expression on various ILC1 subsets and NK cells. Shade, isotype control; black curve, markers above.
(TIF)

S2 Fig Identification of transcriptional factors within CD4^+^ ILC1 subset in human lymphoid organs. (**A**) Representative dot plots depict the expression of transcriptional factor T-bet and Eomes in CD4^+^, CD8^+^ and CD4^-^CD8^-^ ILC1 subsets in various human lymphoid organs. The numbers indicate the percentages of transcriptional factors within each ILC1 subset. (**B** and **C**) Summary data of the expression of T-bet (**B**) and Eomes (**C**) by ILC1 subsets in various lymphoid organs in humans (n = 5).
(TIF)

S3 Fig Phenotypes of CD4^+^ and CD4^-^ ILC1s in peripheral blood. Expression of CD11a, IL-1R1, CD161, HLA-DR, CD38, CD69, CCR6, CXCR3, Ki67, CD95, DR5, caspase 1, caspase 3, CD45RA, CD103 and CD8 on peripheral CD4^+^ and CD4^-^ ILC1s as assessed by flow cytometry (n = 6). The gray shaded curves represent the isotype control.
(TIF)

S4 Fig *In vitro* HIV-1 infection of CD4^+^ T cells. Representative dot plots (**A**) and summarized data (**B**) indicate the p24^+^ ILC1s present in the HIV-1 stock. The numbers (**A**) indicate the percentage of p24^+^ cells in ILC1s. Human PBMCs were infected with HIV-1 (R3A and NL4-3) *in vitro* without or with anti-HIV-1 neutralizing antibody. *p < 0.05 and **p < 0.01, two-tailed paired Student’s *t*-test.
(TIF)

S5 Fig Activation enhances HIV-1 infection in T cells. (**A**) Representative dot plots indicate p24 expression within CD3^+^ T cells present *in vitro* of mock or HIV-1 NL4-3 stock with or without activation (PHA pre-stimulation for 24 hours). (**B**) Summarized data indicate the percentages of p24^+^ cells within CD3^+^ T cells in various conditions. Human PBMCs were first incubated with PHA for 24 hours in the presence of IL-2 (50 IU/ml) and IL-7 (20 ng/ml). The cells were then incubated with HIV/NL4-3 stock or mock stock for additional 4 days. ***p < 0.001, two-tailed paired Student’s *t*-test.
(TIF)

S6 Fig Representative dot plots show expression of the proliferation marker Ki67 in CD4^+^ and CD4^-^ ILC1 subsets from peripheral blood of two healthy donors, two HIV-1 patients and two HAART HIV-1 patients. The numbers indicate percentages of each cell subset.
(TIF)

S7 Fig Correlation analysis between the CD4^+^ ILC1 percentage in CD45^+^ cells and peripheral blood CD4 T cell counts and the CD4/CD8 ratio, respectively. The Spearman rank correlation test is used: *r*, correlation coefficient; *p* values are shown.
(TIF)

S8 Fig Absence of any effect of HIV-1 infection on the expression of caspase 1 and DR5 by ILC1 subsets. (**A**) The representative dot plots depict the expression of caspase 1 on CD4^+^ and CD4^-^ ILC1 subsets in the peripheral blood of various groups. The numbers indicate the percentages of cell subsets. (**B**) Summary data of caspase 1 expression in peripheral blood CD4^+^ and CD4^-^ ILC1s in the HC (n = 15), HIV-1 (n = 27) and HIV-1 plus HAART groups (n = 5). (**C**) Representative dot plots depict DR5 expression on CD4^+^ and CD4^-^ ILC1 subsets in the peripheral blood of various human patients. The numbers indicate percentages of gated cell subsets. (**D**) Summary data of DR5 expression in peripheral blood CD4^+^ and CD4^-^ ILC1s in the HC (n = 6), HIV-1 (n = 6) and HIV-1 plus HAART groups (n = 5). (**B** and **D**) Data represent the mean ± s.e.m. values. **p < 0.01, two-tailed unpaired Student’s *t*-test.
(TIF)

S9 Fig HIV-1 infection leads to CD8 T cell apoptosis. Summary data show the percentage of CD8^+^ T cells expressing active caspase-3 and CD95 from HCs (n = 15), HIV-1-infected patients without HAART (n = 21) and patients with HAART (n = 7). Overall, *p* < 0.05, one-way ANOVA; *p < 0.05, two-tailed unpaired Student’s *t*-test.
(TIF)

S10 Fig The function of ILC-1 in humanized mice with cART and IFNAR blockade. Humanized mice infected with HIV-1 were treated with cART from 4–12 weeks post infection (wpi). From 7 to 10 wpi, the cART-treated mice were injected with α-IFNAR1 antibody or isotype control mIgG2a antibody twice a week. Mice were terminated at 12wpi. **(A)** Representative dot plots show the production of IFN-γ and TNF-α by splenic ILC1s from various groups of humanized mice after stimulation with PMA/ionomycin (n = 5 for each group). Numbers indicate the percentages of cytokine-expressing cell subsets. (**B**) Summarized data of IFN-γ and TNF-α production in response to PMA/ionomycin of splenic ILC1s from mock, HIV-1-infected mice, HIV-1-infected mice with cART plus mIgG2a isotype antibody or α-IFNAR mAb. Data represent the mean ± s.e.m. values. Overall, *p* < 0.05, one-way ANOVA; *p < 0.05 and **p < 0.01, two-tailed unpaired Student’s *t*-test.
(TIF)

## References

[1] Sonnenberg GF, Mjosberg J, Spits H, Artis D. SnapShot: innate lymphoid cells. Immunity. 2013;39(3):622–e1. doi: 10.1016/j.immuni.2013.08.021 . 24012419 10.1016/j.immuni.2013.08.021

[2] Spits H, Cupedo T. Innate lymphoid cells: emerging insights in development, lineage relationships, and function. Annual review of immunology. 2012;30:647–75. doi: 10.1146/annurev-immunol-020711-075053 . 22224763 10.1146/annurev-immunol-020711-075053

[3] Hazenberg MD, Spits H. Human innate lymphoid cells. Blood. 2014;124(5):700–9. doi: 10.1182/blood-2013-11-427781 . 24778151 10.1182/blood-2013-11-427781

[4] Spits H, Artis D, Colonna M, Diefenbach A, Di Santo JP, Eberl G, et al Innate lymphoid cells—a proposal for uniform nomenclature. Nature reviews Immunology. 2013;13(2):145–9. doi: 10.1038/nri3365 . 23348417 10.1038/nri3365

[5] Bernink JH, Peters CP, Munneke M, te Velde AA, Meijer SL, Weijer K, et al Human type 1 innate lymphoid cells accumulate in inflamed mucosal tissues. Nature immunology. 2013;14(3):221–9. Epub 2013/01/22. doi: 10.1038/ni.2534 . 23334791 10.1038/ni.2534

[6] Mjosberg JM, Trifari S, Crellin NK, Peters CP, van Drunen CM, Piet B, et al Human IL-25- and IL-33-responsive type 2 innate lymphoid cells are defined by expression of CRTH2 and CD161. Nature immunology. 2011;12(11):1055–62. doi: 10.1038/ni.2104 . 21909091 10.1038/ni.2104

[7] Magri G, Miyajima M, Bascones S, Mortha A, Puga I, Cassis L, et al Innate lymphoid cells integrate stromal and immunological signals to enhance antibody production by splenic marginal zone B cells. Nature immunology. 2014;15(4):354–64. doi: 10.1038/ni.2830 24562309 10.1038/ni.2830 PMC4005806

[8] Bjorklund AK, Forkel M, Picelli S, Konya V, Theorell J, Friberg D, et al The heterogeneity of human CD127(+) innate lymphoid cells revealed by single-cell RNA sequencing. Nature immunology. 2016;17(4):451–60. Epub 2016/02/16. doi: 10.1038/ni.3368 . 26878113 10.1038/ni.3368

[9] Sonnenberg GF. Transcriptionally defining ILC heterogeneity in humans. Nature immunology. 2016;17(4):351–2. Epub 2016/03/24. doi: 10.1038/ni.3413 . 27002835 10.1038/ni.3413

[10] Simoni Y, Fehlings M, Kloverpris HN, McGovern N, Koo SL, Loh CY, et al Human Innate Lymphoid Cell Subsets Possess Tissue-Type Based Heterogeneity in Phenotype and Frequency. Immunity. 2017;46(1):148–61. doi: 10.1016/j.immuni.2016.11.005 . 27986455 10.1016/j.immuni.2016.11.005 PMC7612935

[11] McKenzie AN, Spits H, Eberl G. Innate lymphoid cells in inflammation and immunity. Immunity. 2014;41(3):366–74. doi: 10.1016/j.immuni.2014.09.006 . 25238094 10.1016/j.immuni.2014.09.006

[12] Sonnenberg GF, Artis D. Innate lymphoid cells in the initiation, regulation and resolution of inflammation. Nature medicine. 2015;21(7):698–708. Epub 2015/06/30. doi: 10.1038/nm.3892 26121198 10.1038/nm.3892 PMC4869856

[13] Zook EC, Kee BL. Development of innate lymphoid cells. Nature immunology. 2016;17(7):775–82. Epub 2016/06/22. doi: 10.1038/ni.3481 . 27328007 10.1038/ni.3481

[14] Xu H, Wang X, Liu DX, Moroney-Rasmussen T, Lackner AA, Veazey RS. IL-17-producing innate lymphoid cells are restricted to mucosal tissues and are depleted in SIV-infected macaques. Mucosal immunology. 2012;5(6):658–69. doi: 10.1038/mi.2012.39 22669579 10.1038/mi.2012.39 PMC3702374

[15] Li H, Richert-Spuhler LE, Evans TI, Gillis J, Connole M, Estes JD, et al Hypercytotoxicity and Rapid Loss of NKp44+ Innate Lymphoid Cells during Acute SIV Infection. PLoS pathogens. 2014;10(12):e1004551 doi: 10.1371/journal.ppat.1004551 25503264 10.1371/journal.ppat.1004551 PMC4263758

[16] Kloverpris HN, Kazer SW, Mjosberg J, Mabuka JM, Wellmann A, Ndhlovu Z, et al Innate Lymphoid Cells Are Depleted Irreversibly during Acute HIV-1 Infection in the Absence of Viral Suppression. Immunity. 2016;44(2):391–405. Epub 2016/02/07. doi: 10.1016/j.immuni.2016.01.006 . 26850658 10.1016/j.immuni.2016.01.006 PMC6836297

[17] Zhang Z, Cheng L, Zhao J, Li G, Zhang L, Chen W, et al Plasmacytoid dendritic cells promote HIV-1-induced group 3 innate lymphoid cell depletion. The Journal of clinical investigation. 2015;125(9):3692–703. Epub 2015/08/25. doi: 10.1172/JCI82124 26301812 10.1172/JCI82124 PMC4588300

[18] Kramer B, Goeser F, Lutz P, Glassner A, Boesecke C, Schwarze-Zander C, et al Compartment-specific distribution of human intestinal innate lymphoid cells is altered in HIV patients under effective therapy. PLoS pathogens. 2017;13(5):e1006373 doi: 10.1371/journal.ppat.1006373 28505204 10.1371/journal.ppat.1006373 PMC5444854

[19] Xu H, Wang X, Lackner AA, Veazey RS. Type 3 innate lymphoid cell depletion is mediated by TLRs in lymphoid tissues of simian immunodeficiency virus-infected macaques. FASEB journal: official publication of the Federation of American Societies for Experimental Biology. 2015;29(12):5072–80. Epub 2015/08/19. doi: 10.1096/fj.15-276477 26283536 10.1096/fj.15-276477 PMC4653054

[20] Roan F, Stoklasek TA, Whalen E, Molitor JA, Bluestone JA, Buckner JH, et al CD4+ Group 1 Innate Lymphoid Cells (ILC) Form a Functionally Distinct ILC Subset That Is Increased in Systemic Sclerosis. Journal of immunology. 2016;196(5):2051–62. Epub 2016/01/31. doi: 10.4049/jimmunol.1501491 26826243 10.4049/jimmunol.1501491 PMC4761490

[21] Bonsignori M, Montefiori DC, Wu X, Chen X, Hwang KK, Tsao CY, et al Two distinct broadly neutralizing antibody specificities of different clonal lineages in a single HIV-1-infected donor: implications for vaccine design. Journal of virology. 2012;86(8):4688–92. Epub 2012/02/04. doi: 10.1128/JVI.07163-11 22301150 10.1128/JVI.07163-11 PMC3318651

[22] Zhang L, Kovalev GI, Su L. HIV-1 infection and pathogenesis in a novel humanized mouse model. Blood. 2007;109(7):2978–81. doi: 10.1182/blood-2006-07-033159 17132723 10.1182/blood-2006-07-033159 PMC1852218

[23] Shultz LD, Brehm MA, Garcia JV, Greiner DL. Humanized mice for immune system investigation: progress, promise and challenges. Nature Reviews Immunology. 2012;12(11):786–98. doi: 10.1038/nri3311 23059428 10.1038/nri3311 PMC3749872

[24] Jamieson BD, Zack JA. In vivo pathogenesis of a human immunodeficiency virus type 1 reporter virus. Journal of virology. 1998;72(8):6520–6. Epub 1998/07/11. 9658095 10.1128/jvi.72.8.6520-6526.1998 PMC109821

[25] Bosinger SE, Utay NS. Type I interferon: understanding its role in HIV pathogenesis and therapy. Current HIV/AIDS reports. 2015;12(1):41–53. Epub 2015/02/11. doi: 10.1007/s11904-014-0244-6 . 25662992 10.1007/s11904-014-0244-6

[26] Cheng L, Ma J, Li J, Li D, Li G, Li F, et al Blocking type I interferon signaling enhances T cell recovery and reduces HIV-1 reservoirs. The Journal of clinical investigation. 2017;127(1):269–79. Epub 2016/12/13. doi: 10.1172/JCI90745 27941247 10.1172/JCI90745 PMC5199717

[27] Zhen A, Rezek V, Youn C, Lam B, Chang N, Rick J, et al Targeting type I interferon-mediated activation restores immune function in chronic HIV infection. The Journal of clinical investigation. 2017;127(1):260–8. Epub 2016/12/13. doi: 10.1172/JCI89488 27941243 10.1172/JCI89488 PMC5199686

[28] Cheng L, Yu H, Li G, Li F, Ma J, Li J, et al Type I interferons suppress viral replication but contribute to T cell depletion and dysfunction during chronic HIV-1 infection. JCI Insight. 2017;2(12). doi: 10.1172/jci.insight.94366 28614789 10.1172/jci.insight.94366 PMC5470878

[29] Bernink JH, Mjosberg J, Spits H. Human ILC1: To Be or Not to Be. Immunity. 2017;46(5):756–7. doi: 10.1016/j.immuni.2017.05.001 28514676 10.1016/j.immuni.2017.05.001 PMC5441993

[30] Simoni Y, Newell EW. Toward Meaningful Definitions of Innate-Lymphoid-Cell Subsets. Immunity. 2017;46(5):760–1. doi: 10.1016/j.immuni.2017.04.026 . 28514678 10.1016/j.immuni.2017.04.026

[31] Zhang C, Xin H, Zhang W, Yazaki PJ, Zhang Z, Le K, et al CD5 Binds to Interleukin-6 and Induces a Feed-Forward Loop with the Transcription Factor STAT3 in B Cells to Promote Cancer. Immunity. 2016;44(4):913–23. doi: 10.1016/j.immuni.2016.04.003 27096320 10.1016/j.immuni.2016.04.003 PMC4844010

[32] Roan F, Ziegler SF. Human Group 1 Innate Lymphocytes Are Negative for Surface CD3epsilon but Express CD5. Immunity. 2017;46(5):758–9. doi: 10.1016/j.immuni.2017.04.024 28514677 10.1016/j.immuni.2017.04.024 PMC5503209

[33] Vely F, Barlogis V, Vallentin B, Neven B, Piperoglou C, Ebbo M, et al Evidence of innate lymphoid cell redundancy in humans. Nature immunology. 2016;17(11):1291–9. Epub 2016/10/21. doi: 10.1038/ni.3553 27618553 10.1038/ni.3553 PMC5074366

[34] Bernink JH, Krabbendam L, Germar K, de Jong E, Gronke K, Kofoed-Nielsen M, et al Interleukin-12 and -23 Control Plasticity of CD127(+) Group 1 and Group 3 Innate Lymphoid Cells in the Intestinal Lamina Propria. Immunity. 2015;43(1):146–60. Epub 2015/07/19. doi: 10.1016/j.immuni.2015.06.019 . 26187413 10.1016/j.immuni.2015.06.019

[35] Lundgren JD, Babiker AG, Gordin F, Emery S, Grund B, Sharma S, et al Initiation of Antiretroviral Therapy in Early Asymptomatic HIV Infection. The New England journal of medicine. 2015;373(9):795–807. Epub 2015/07/21. doi: 10.1056/NEJMoa1506816 26192873 10.1056/NEJMoa1506816 PMC4569751

[36] Smaill F. In early HIV infection, immediate vs deferred antiretroviral therapy reduced serious illnesses at 3 years. Annals of internal medicine. 2015;163(12):JC4 Epub 2015/12/17. doi: 10.7326/ACPJC-2015-163-12-004 . 26666806 10.7326/ACPJC-2015-163-12-004

