# Supplementary material for: Infection and depletion of CD4+ group-1 innate lymphoid cells by HIV-1 via type-I interferon pathway
Source: PLoS Pathog. 2018 Jan 5;14(1):e1006819. doi: 10.1371/journal.ppat.1006819 (PMC5773236; doi:10.1371/journal.ppat.1006819)
Supplement: S1 Table — (DOCX) [file ppat.1006819.s001.docx]

**S1 Table Characteristics of the study participants**

| Groups | HC | HIV-1 | HIV-1  +HAART |
| --- | --- | --- | --- |
| No. of cases | 26 | 30 | 12 |
| Age (y) | 37 (19–57) | 45 (25–56) | 41 (29–54) |
| Sex (m/f) | 18/8 | 18/12 | 9/3 |
| CD4 T-cell count (cells/μl) | NA | 333 (69–493) | 426 (291–1178) |
| CD8 T-cell count (cells/μl) | NA | 876 (376–3216) | 931 (437–2064) |
| CD4/CD8 ratio | NA | 0.28 (0.08–0.59) | 0.57 (0.26–1.03) |
| HIV-1 load (10^4^ copies/ml) | NA | 7.58 (0.22–460) | <20 |
| Infection route (s/b/nd)^*^ | NA | 18/9/3 | 8/3/1 |

HC, Healthy controls; NA, not applicable; Data shown are the median (range). ^*^b, blood; s, sex; nd, not determined.
