# Supplementary figures and images for: Infection and depletion of CD4+ group-1 innate lymphoid cells by HIV-1 via type-I interferon pathway
Source: PLoS Pathog. 2018 Jan 5;14(1):e1006819. doi: 10.1371/journal.ppat.1006819 (PMC5773236; doi:10.1371/journal.ppat.1006819)

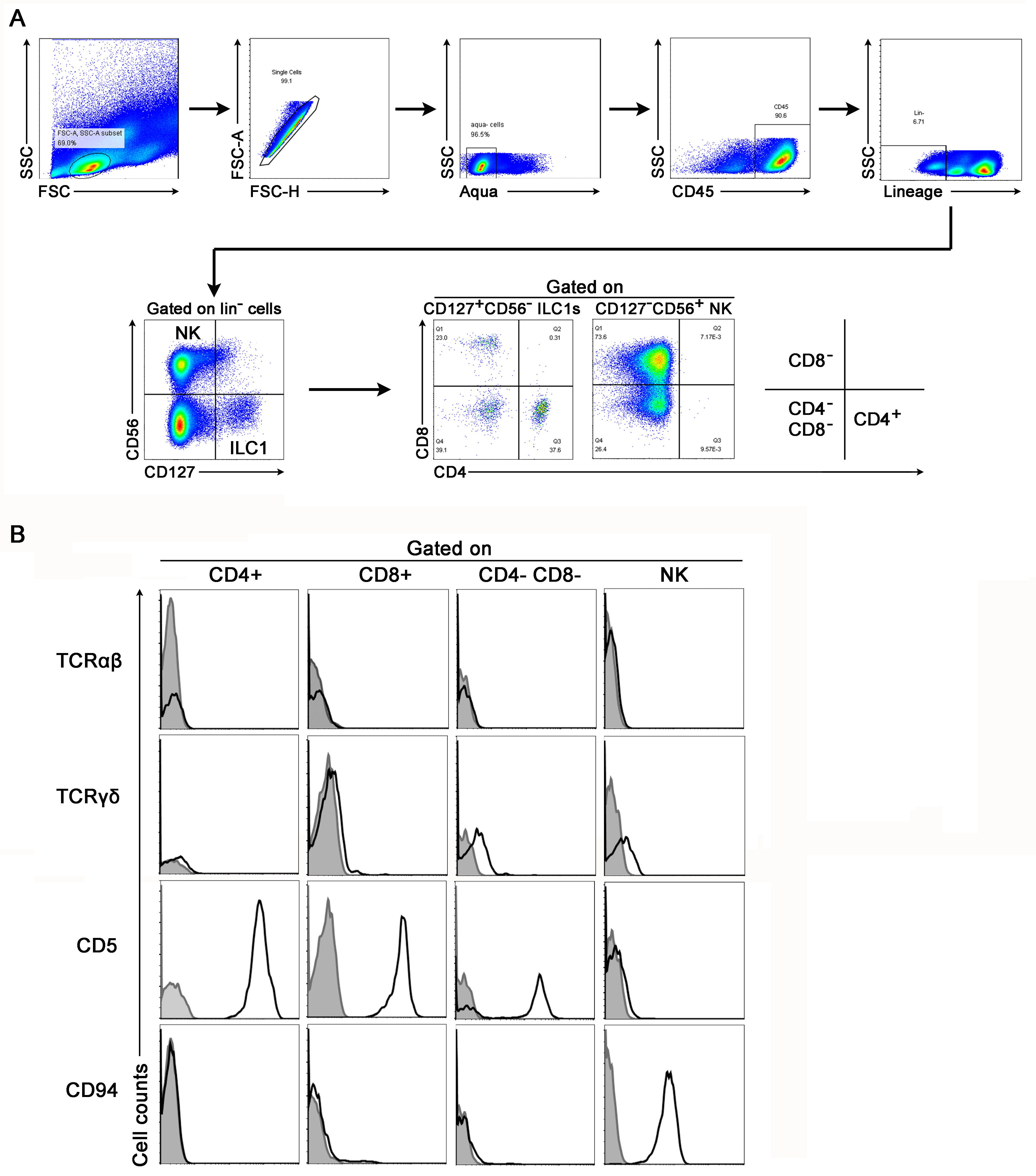

Supplement: S1 Fig — (A) Representative dot plots identify ILC1 subsets. After gating on lymphocytes (FSC-SSC), singlets, and live CD45+ cells, the cells that remained were identified as lineage-CD127-CD56+ NK cells and lineage-CD127+CD56- total ILC1 cells. Based on CD4 and CD8 expression, ILC1s were further divided into CD4+CD8-, CD4-CD8+ and CD4-CD8- ILC1s, while the NK cells did not contain any CD4+CD8- cell subpopulations. The lineage markers included CD3, CD14, CD16, CD19, CD123, CD11c, CD34, CD117 (excluding ILC3) and CD294 (CRTH2, excluding ILC2). The numbers indicate the percentage of cell subsets. (B) A representative histogram shows the expression of TCRαβ, TCRγδ, CD5 and CD94 expression on various ILC1 subsets and NK cells. Shade, isotype control; black curve, markers above. (TIF) [file ppat.1006819.s002.tif]

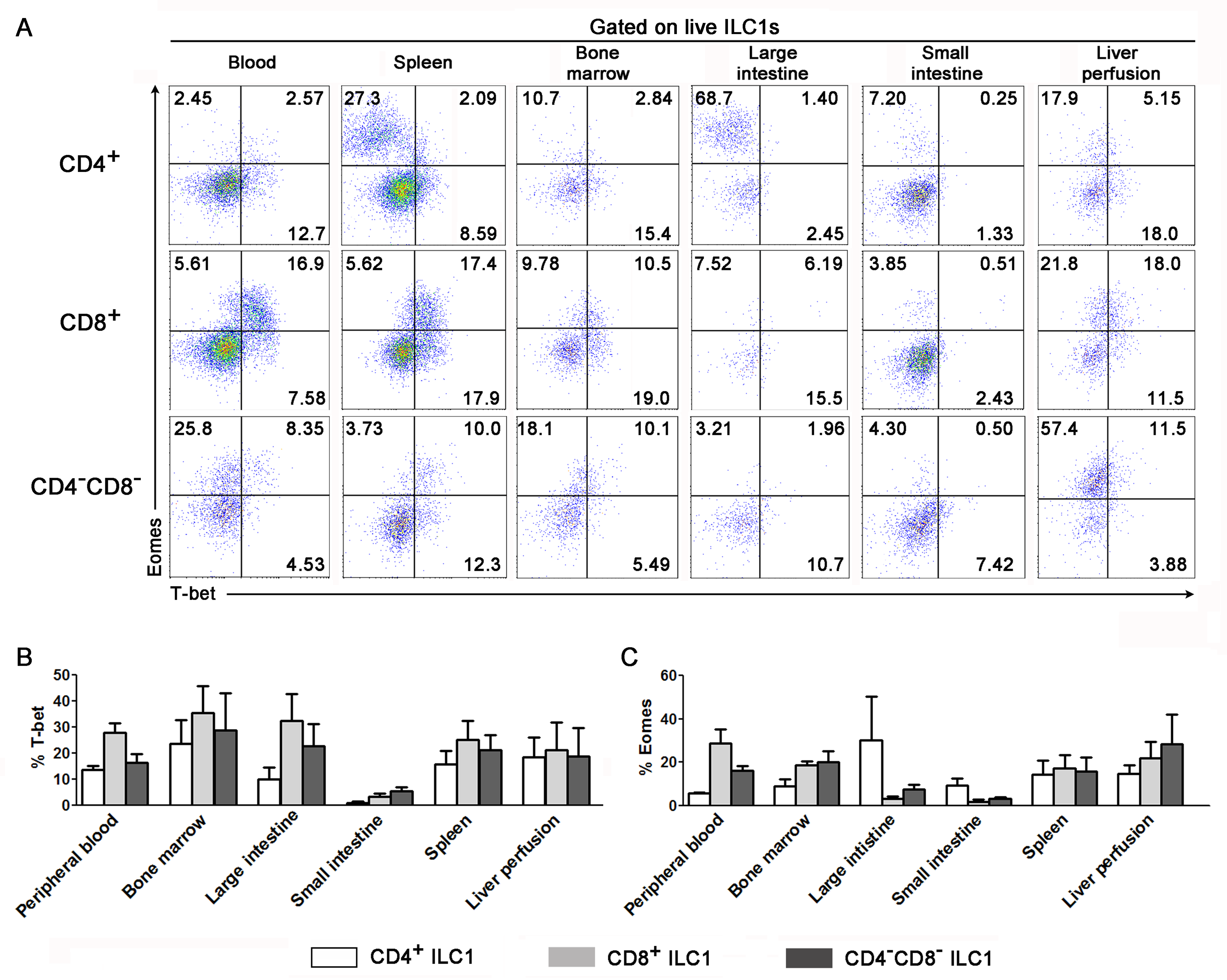

Supplement: S2 Fig — (A) Representative dot plots depict the expression of transcriptional factor T-bet and Eomes in CD4+, CD8+ and CD4-CD8- ILC1 subsets in various human lymphoid organs. The numbers indicate the percentages of transcriptional factors within each ILC1 subset. (B and C) Summary data of the expression of T-bet (B) and Eomes (C) by ILC1 subsets in various lymphoid organs in humans (n = 5). (TIF) [file ppat.1006819.s003.tif]

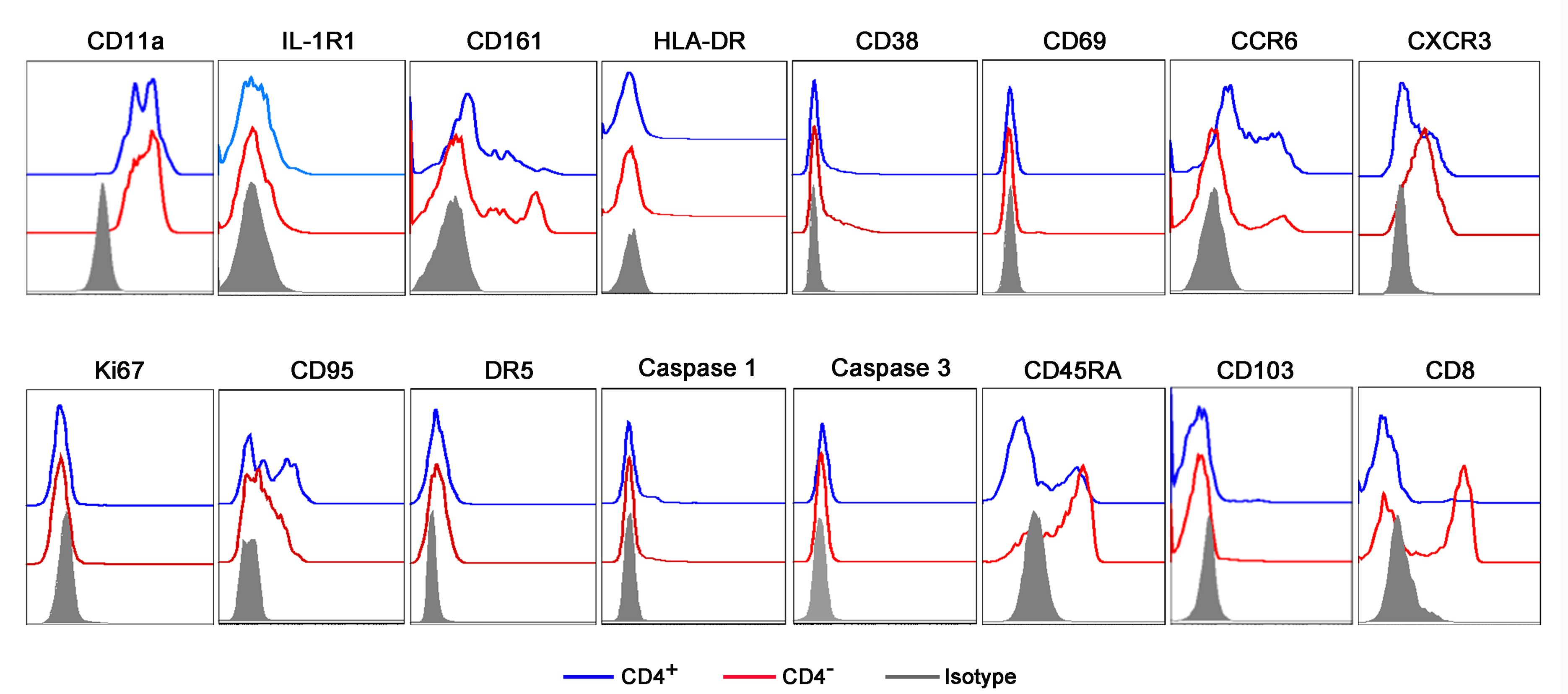

Supplement: S3 Fig — Expression of CD11a, IL-1R1, CD161, HLA-DR, CD38, CD69, CCR6, CXCR3, Ki67, CD95, DR5, caspase 1, caspase 3, CD45RA, CD103 and CD8 on peripheral CD4+ and CD4- ILC1s as assessed by flow cytometry (n = 6). The gray shaded curves represent the isotype control. (TIF) [file ppat.1006819.s004.tif]

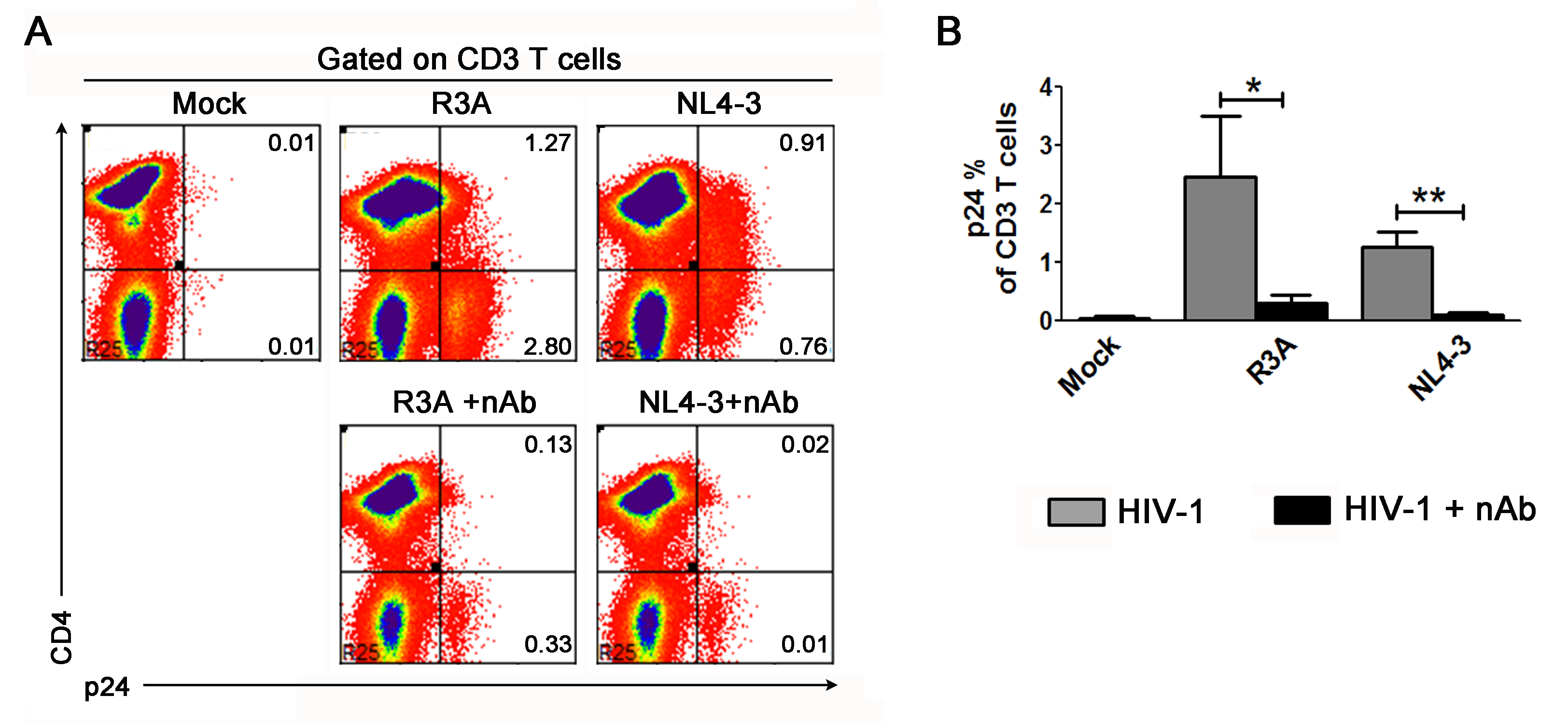

Supplement: S4 Fig — Representative dot plots (A) and summarized data (B) indicate the p24+ ILC1s present in the HIV-1 stock. The numbers (A) indicate the percentage of p24+ cells in ILC1s. Human PBMCs were infected with HIV-1 (R3A and NL4-3) in vitro without or with anti-HIV-1 neutralizing antibody. *p < 0.05 and **p < 0.01, two-tailed paired Student’s t-test. (TIF) [file ppat.1006819.s005.tif]

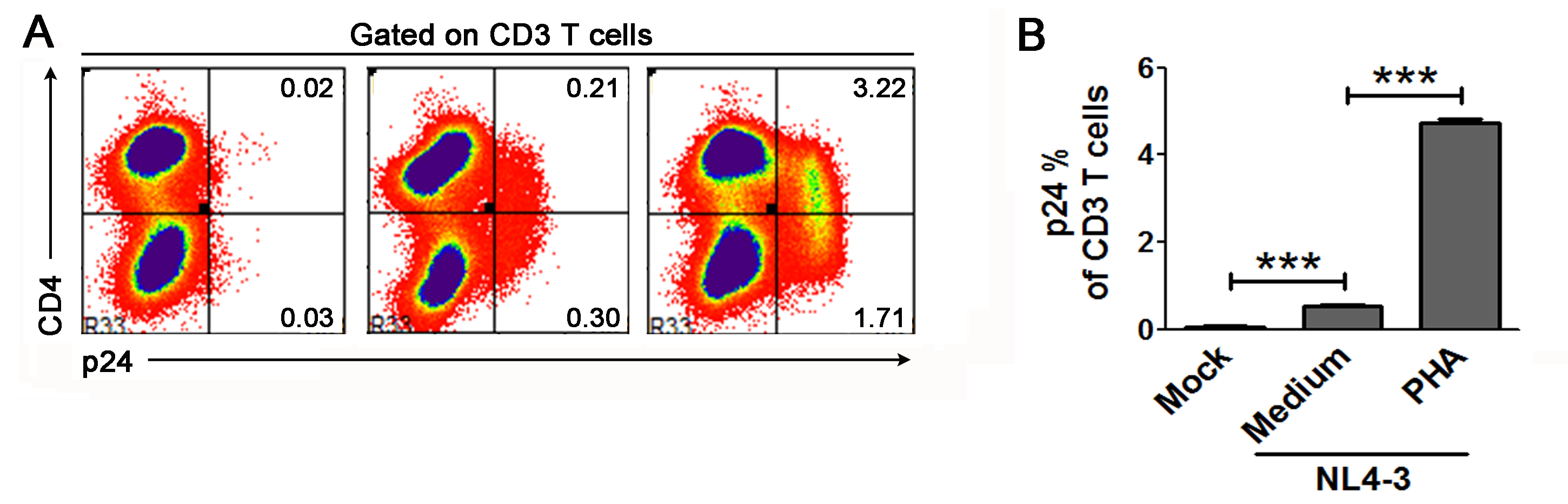

Supplement: S5 Fig — (A) Representative dot plots indicate p24 expression within CD3+ T cells present in vitro of mock or HIV-1 NL4-3 stock with or without activation (PHA pre-stimulation for 24 hours). (B) Summarized data indicate the percentages of p24+ cells within CD3+ T cells in various conditions. Human PBMCs were first incubated with PHA for 24 hours in the presence of IL-2 (50 IU/ml) and IL-7 (20 ng/ml). The cells were then incubated with HIV/NL4-3 stock or mock stock for additional 4 days. ***p < 0.001, two-tailed paired Student’s t-test. (TIF) [file ppat.1006819.s006.tif]

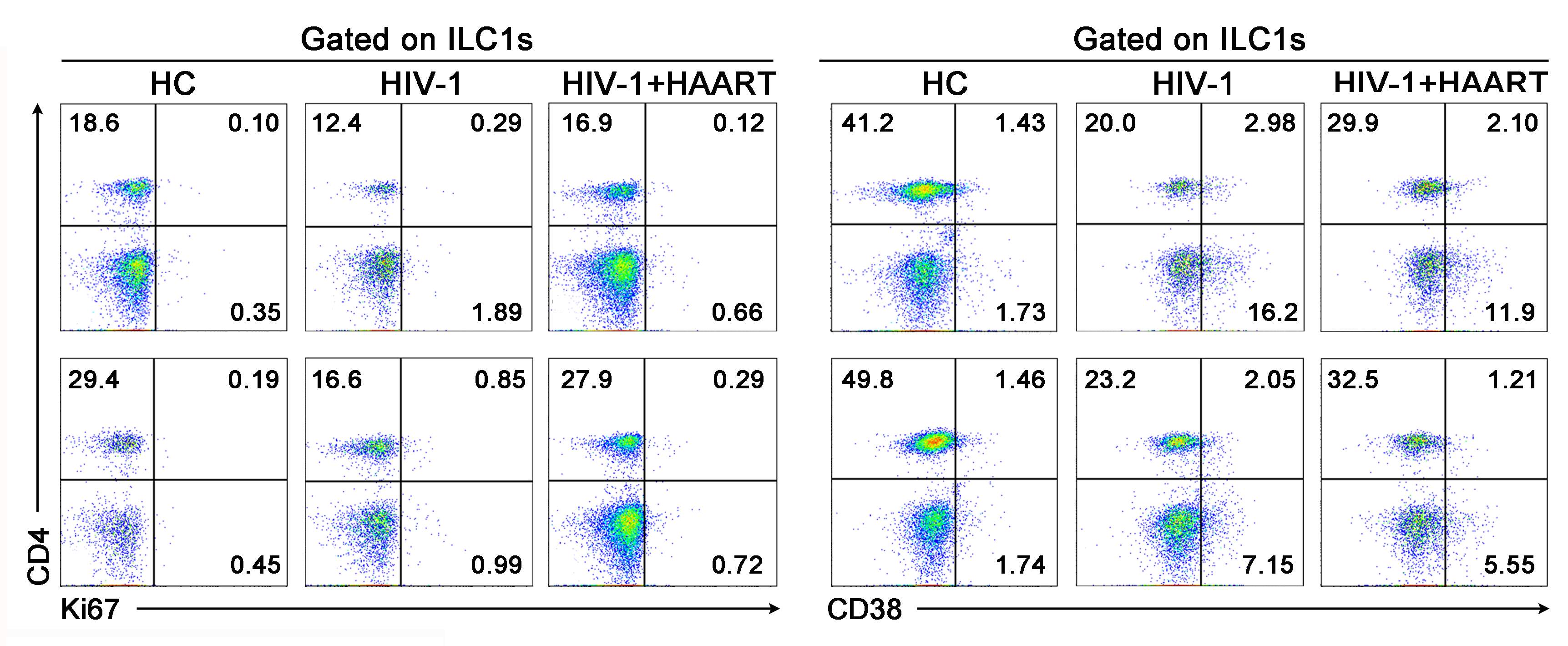

Supplement: S6 Fig — The numbers indicate percentages of each cell subset. (TIF) [file ppat.1006819.s007.tif]

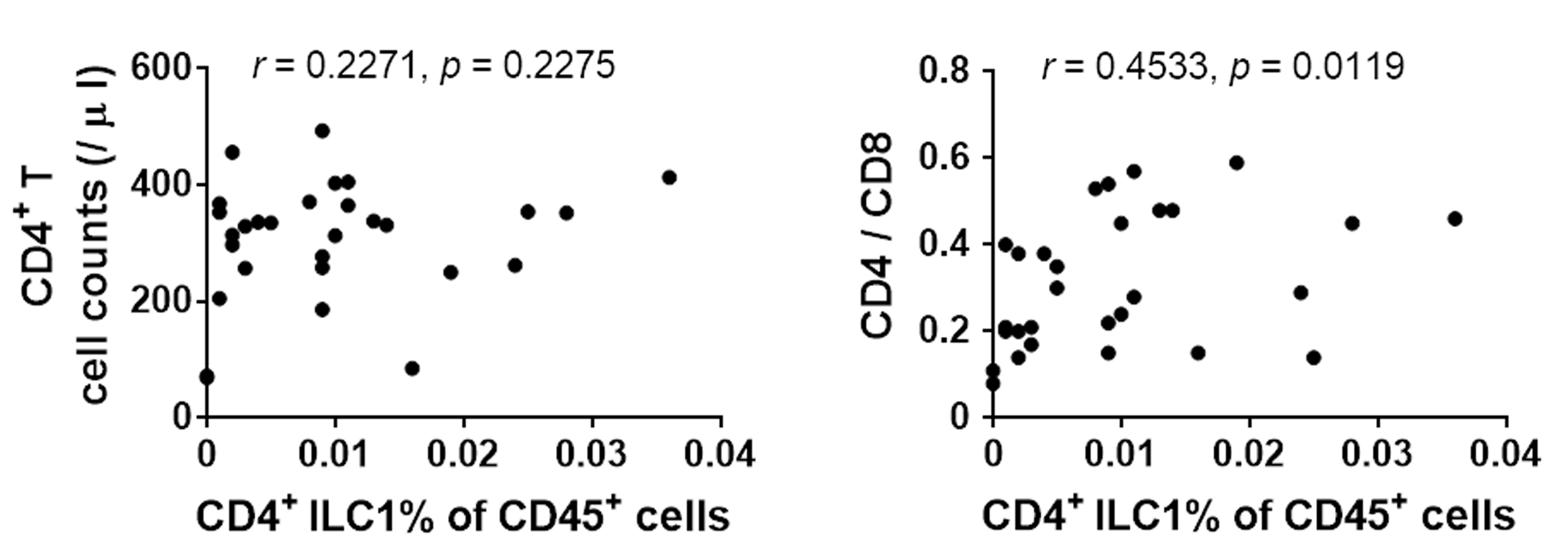

Supplement: S7 Fig — The Spearman rank correlation test is used: r, correlation coefficient; p values are shown. (TIF) [file ppat.1006819.s008.tif]

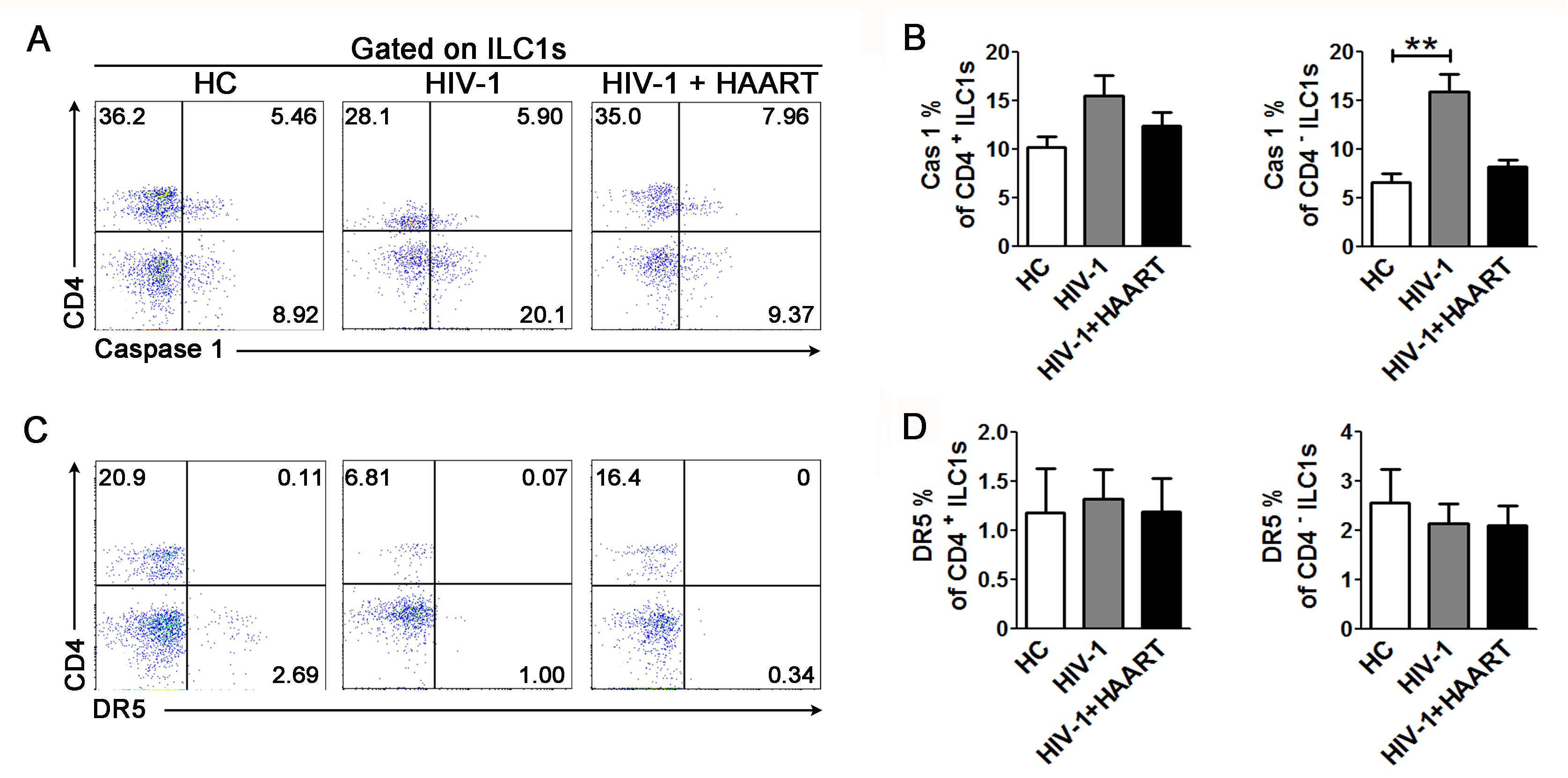

Supplement: S8 Fig — (A) The representative dot plots depict the expression of caspase 1 on CD4+ and CD4- ILC1 subsets in the peripheral blood of various groups. The numbers indicate the percentages of cell subsets. (B) Summary data of caspase 1 expression in peripheral blood CD4+ and CD4- ILC1s in the HC (n = 15), HIV-1 (n = 27) and HIV-1 plus HAART groups (n = 5). (C) Representative dot plots depict DR5 expression on CD4+ and CD4- ILC1 subsets in the peripheral blood of various human patients. The numbers indicate percentages of gated cell subsets. (D) Summary data of DR5 expression in peripheral blood CD4+ and CD4- ILC1s in the HC (n = 6), HIV-1 (n = 6) and HIV-1 plus HAART groups (n = 5). (B and D) Data represent the mean ± s.e.m. values. **p < 0.01, two-tailed unpaired Student’s t-test. (TIF) [file ppat.1006819.s009.tif]

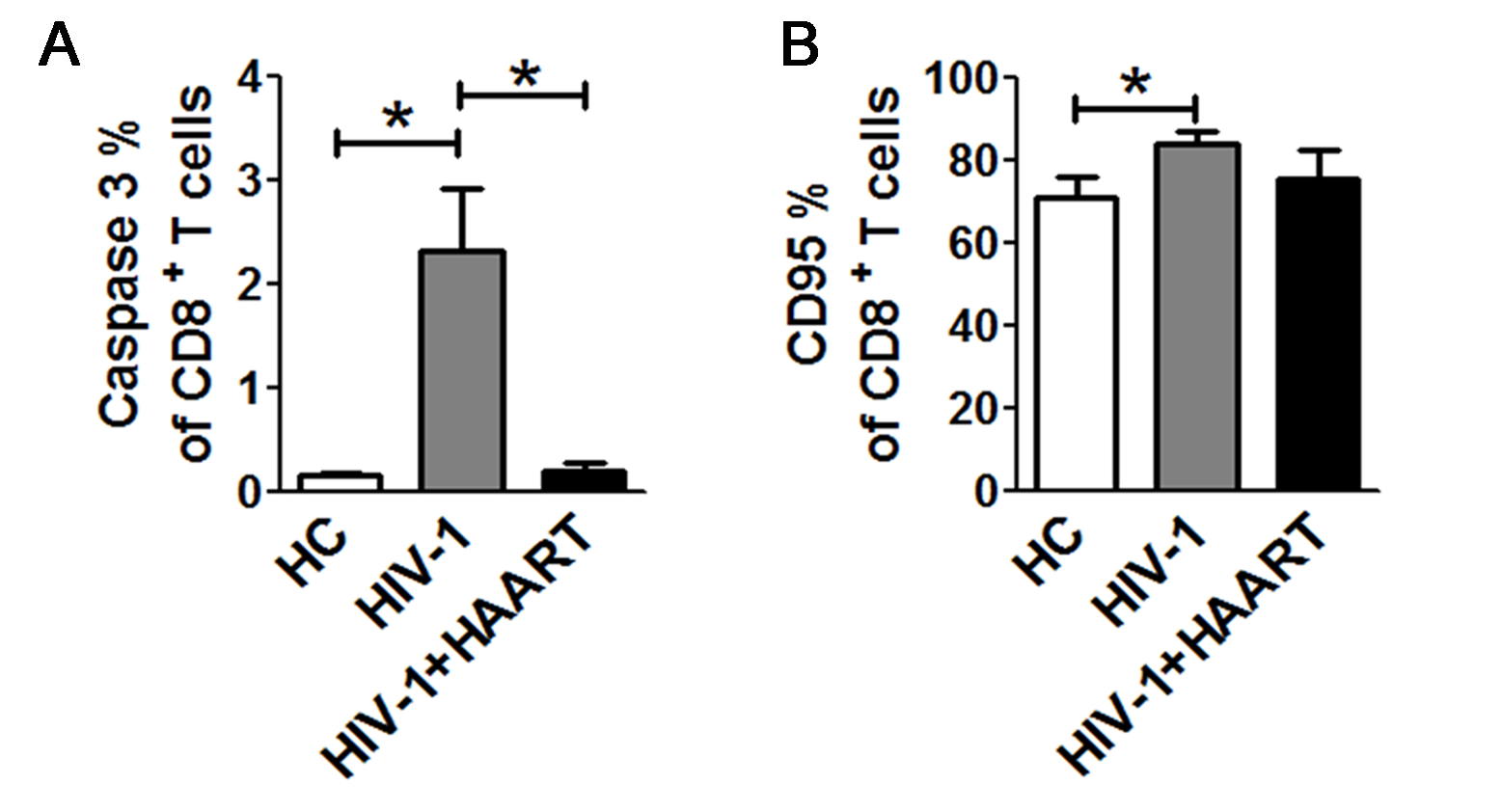

Supplement: S9 Fig — Summary data show the percentage of CD8+ T cells expressing active caspase-3 and CD95 from HCs (n = 15), HIV-1-infected patients without HAART (n = 21) and patients with HAART (n = 7). Overall, p < 0.05, one-way ANOVA; *p < 0.05, two-tailed unpaired Student’s t-test. (TIF) [file ppat.1006819.s010.tif]

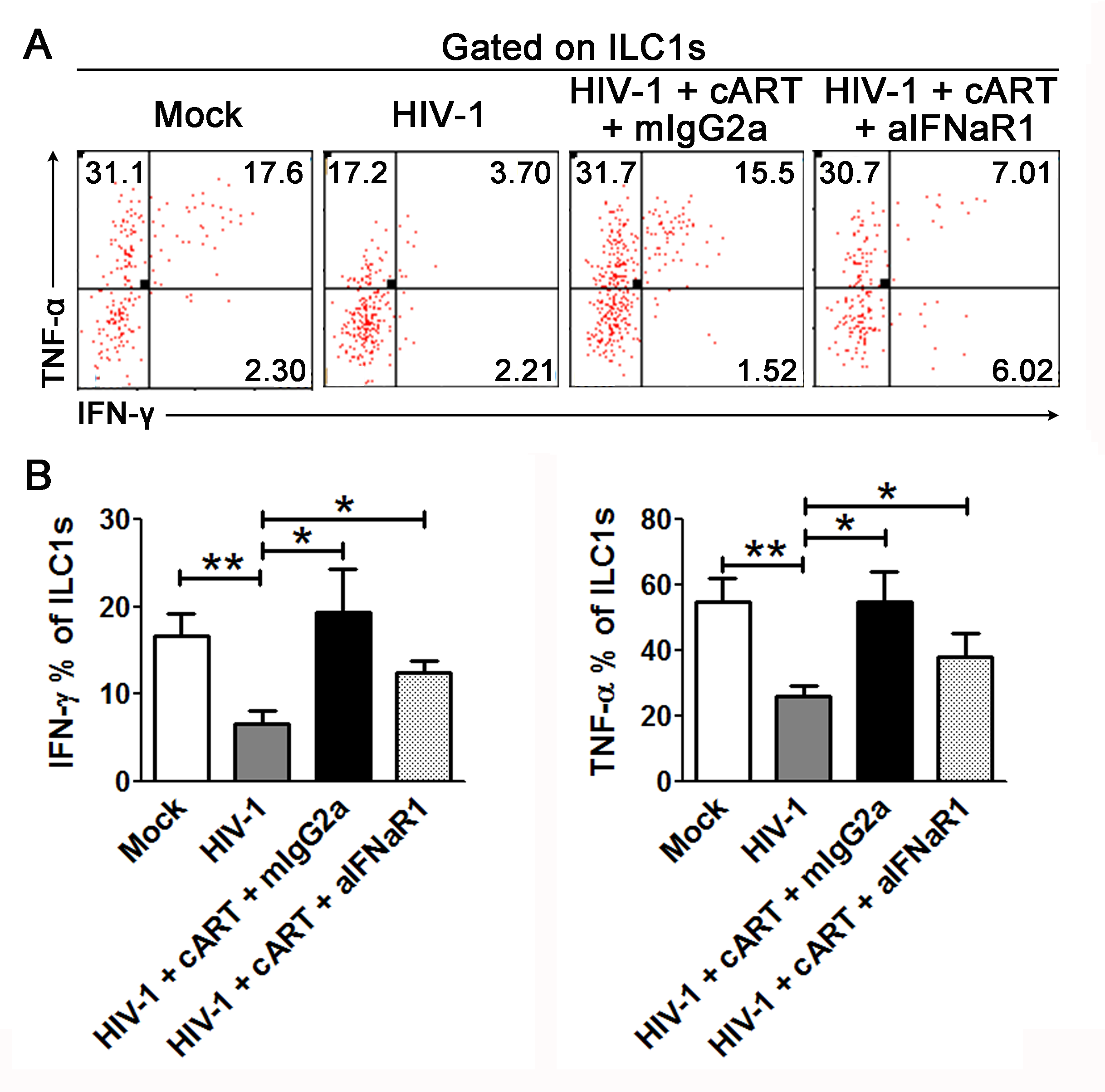

Supplement: S10 Fig — Humanized mice infected with HIV-1 were treated with cART from 4–12 weeks post infection (wpi). From 7 to 10 wpi, the cART-treated mice were injected with α-IFNAR1 antibody or isotype control mIgG2a antibody twice a week. Mice were terminated at 12wpi. (A) Representative dot plots show the production of IFN-γ and TNF-α by splenic ILC1s from various groups of humanized mice after stimulation with PMA/ionomycin (n = 5 for each group). Numbers indicate the percentages of cytokine-expressing cell subsets. (B) Summarized data of IFN-γ and TNF-α production in response to PMA/ionomycin of splenic ILC1s from mock, HIV-1-infected mice, HIV-1-infected mice with cART plus mIgG2a isotype antibody or α-IFNAR mAb. Data represent the mean ± s.e.m. values. Overall, p < 0.05, one-way ANOVA; *p < 0.05 and **p < 0.01, two-tailed unpaired Student’s t-test. (TIF) [file ppat.1006819.s011.tif]
